# A new, widespread venomous mammal species: hemolytic activity of *Sorex araneus* venom is similar to that of *Neomys fodiens* venom

**DOI:** 10.1186/s40851-022-00191-5

**Published:** 2022-06-07

**Authors:** Krzysztof Kowalski, Paweł Marciniak, Leszek Rychlik

**Affiliations:** 1 Department of Vertebrate Zoology and Ecology, Institute of Biology, Faculty of Biological and Veterinary Sciences, Nicolaus Copernicus University, Lwowska 1, 87-100 Toruń, Poland; 2 Department of Animal Physiology and Developmental Biology, Institute of Experimental Biology, Faculty of Biology, Adam Mickiewicz University, Uniwersytetu Poznańskiego 6, 61-614 Poznań, Poland; 3 Department of Systematic Zoology, Institute of Environmental Biology, Faculty of Biology, Adam Mickiewicz University, Uniwersytetu Poznańskiego 6, 61-614 Poznań, Poland

**Keywords:** cytotoxicity, eulipotyphlans, hemolysis, mammalian venom, prey hunting, shrews, venom evolution

## Abstract

**Background:**

Venom production has evolved independently many times in the animal kingdom, although it is rare among mammals. Venomous shrews produce toxins in their salivary glands and use their venoms to hunt and store prey. Thus far, the toxicity and composition of shrew venoms have been studied only in two shrew species: the northern short-tailed shrew, *Blarina brevicauda*, and the Eurasian water shrew, *Neomys fodiens*. Venom of *N. fodiens* has potent paralytic activity which enables hunting and storing prey in a comatose state. Here, we assayed the hemolytic effects of extracts from salivary glands of *N. fodiens* and the common shrew, *Sorex araneus*, in erythrocytes of *Pelophylax* sp. frogs. We identified toxins in shrew venom by high-performance liquid chromatography coupled to tandem mass spectrometry.

**Results:**

Our results prove, confirming a suggestion made four centuries ago, that *S. araneus* is venomous. We also provide the first experimental evidence that shrew venoms produce potent hemolysis in frog erythrocytes. We found significant concentration-dependent effects of venoms of *N. fodiens* and *S. araneus* on hemolysis of red blood cells evaluated as hemoglobin release. Treatment of erythrocytes with *N. fodiens* venom at concentrations of 1.0 and 0.5 mg/ml and with *S. araneus* venom at concentration of 1.0 mg/ml caused an increased release of hemoglobin. Our findings confirm that hemolytic effects of *N. fodiens* venom are stronger than those produced by *S. araneus* venom. We identified four toxins in the venom of *N. fodiens*: proenkephalin, phospholipase A_2_ (PLA_2_), a disintegrin and metalloproteinase domain-containing protein (ADAM) and lysozyme C, as well as a non-toxic hyaluronidase. In the venom of *S. araneus* we found five toxins: proenkephalin, kallikrein 1-related peptidase, beta-defensin, ADAM and lysozyme C. PLA_2_ and ADAMs are likely to produce hemolysis in frog erythrocytes.

**Conclusions:**

Our results clearly show that shrew venoms possess hemolytic action that may allow them to hunt larger prey. Since a member of the numerous genus *Sorex* is venomous, it is likely that venom production among shrews and other eulipotyphlans may be more widespread than it has previously been assumed.

**Supplementary Information:**

The online version contains supplementary material available at 10.1186/s40851-022-00191-5.

## Background

The use of venom for prey acquisition, predator deterrence and competition is widespread in the animal kingdom [1, 2]. However, in mammals venom production is rare and restricted to the members of four extant orders, the monotremes, eulipotyphlans, chiropterans and primates [3–6]. Most venomous mammals belong to the order Eulipotyphla [7], which consists of moles, shrews, solenodons and hedgehogs [8], but venomous representatives have been found only among shrews and solenodons thus far [7, 9–15].

Venomous eulipotyphlans use their venoms to capture and immobilize prey for long-term storage [4, 6, 16]. Such food hoarding can save energy and time spent on foraging and capturing prey, and minimize the risk of predation [7, 16]. Thus, prey hoarding brings considerable benefits to eulipotyphlans, especially the Soricinae shrews which, due to extremely high metabolic rate, have to consume large amounts of food to meet their energetic demands [17, 18].

**Fig. 1:**
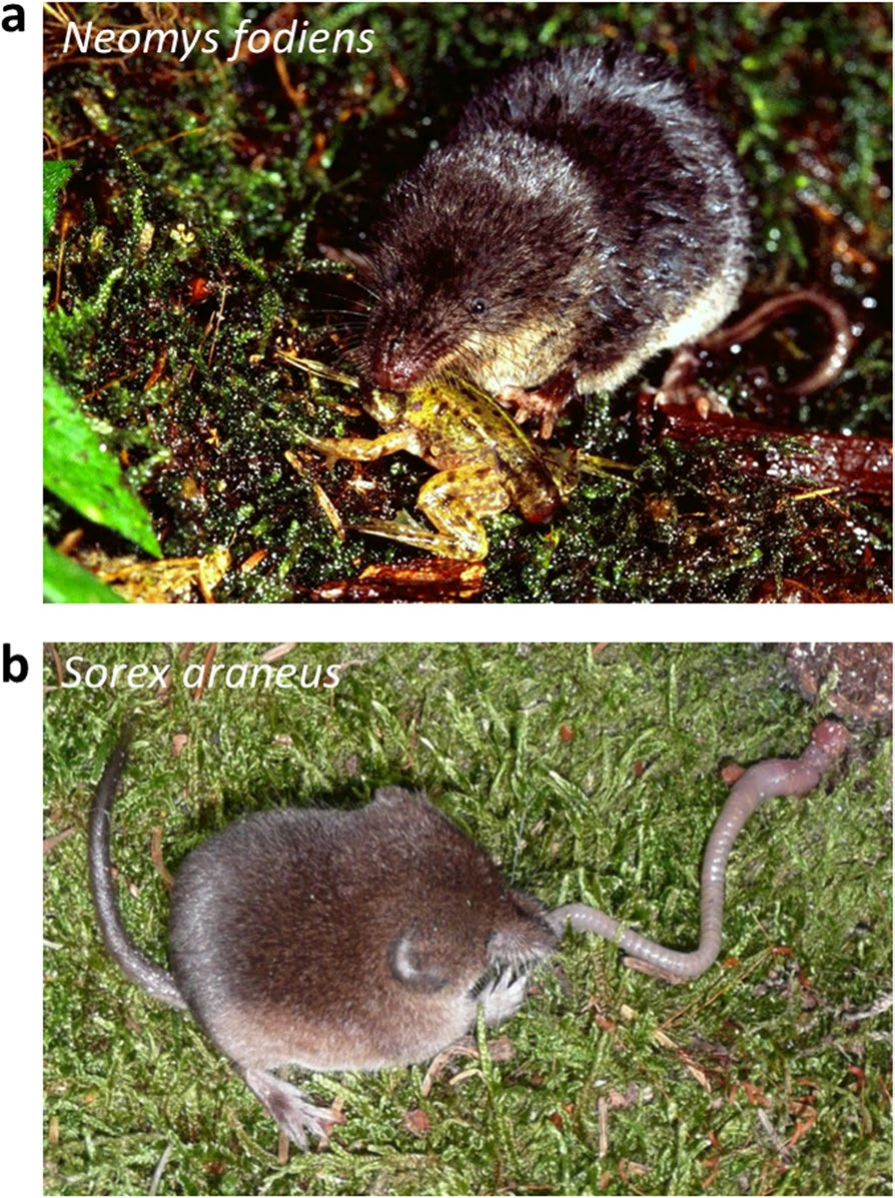
Venomous shrew species (**a**) The Eurasian water shrew, *Neomys fodiens*. (**b**) The common shrew, *Sorex araneus*. Photos: M. Mounier (**a**) and S. von Merten (**b**)

Studies on the toxicity and composition of eulipotyphlan venoms are scarce. Among shrews, only venoms of the northern short-tailed shrew, *Blarina brevicauda*, and the Eurasian water shrew, *Neomys fodiens* (Fig. 1a), have been analyzed thus far [9, 12, 13, 15, 19]. The venom of *B. brevicauda* has proteolytic and hypotensive activity [12], while venom of *N. fodiens* is known to produce potent paralytic effects [9, 13]. Pucek [9] was the first to observe the paralysis of the limbs and posterior part of the body in experimental animals after treatment with the crude extract from salivary glands of *N. fodiens*. Recently, Kowalski et al. [13] confirmed strong neurotoxic and lower cardioinhibitory activity of the venom of *N. fodiens*.

Animal venoms usually are of complex nature in both composition and toxicity [20–22]. For instance, venoms of many taxa such as snakes, scorpions, marine snails and jellyfish produce potent cytotoxic effects [23–30] which enable them to rapidly subdue prey and avoid retaliatory injuries, especially if the attacked prey is large or potentially dangerous [7]. Proteases, phospholipases, and cytolytic or hemolytic agents are among the primary molecules responsible for the venom cytotoxicity [26]. Snake venom metalloproteinases (SVMPs) are known to provide hemotoxic activity of snake venoms, particularly in species from the Viperidae family and Crotalinae subfamily [31, 32]. Because eulipotyphlans often prey upon larger prey, some of which may be even larger than themselves [11, 16, 33–35], it is likely that their venoms also display cytotoxicity, though it has not yet been verified.

Our recent study confirmed that *N. fodiens* venom contains a phospholipase A_2_ (PLA_2_) [13]. PLA_2_ is known for varied toxic activities, including hemolysis [24, 25, 27, 28]. Thus, the presence of PLA_2_ in the venom of *N. fodiens* might be indicative of hemolytic effects of its venom. With this in mind, in the present study we aimed to determine the hemolytic activity of salivary gland extracts of *N. fodiens* and, for comparison, the common shrew, *Sorex araneus* (Fig. 1b) hitherto considered non-venomous. To achieve our goal we performed physiological bioassays on the erythrocytes of *Pelophylax* sp. frogs. We hypothesized that *N. fodiens* venom would produce potent and concentration-dependent hemolysis in frog erythrocytes, while saliva of *S. araneus* [13] would not present any hemolytic effects. Because only few venom proteins have been found in shrew venoms so far [12–15, 19], we also aimed to identify new toxins in the venom of *N. fodiens* and non-toxic molecules in the saliva of *S. araneus*. To do so, we performed proteomic analyses to determine protein content in both, the whole extract from venom glands of both shrew species and in fractions separated by high-performance liquid chromatography.

## Results

### Shrew venoms produce hemolysis in frog erythrocytes

**Fig. 2:**
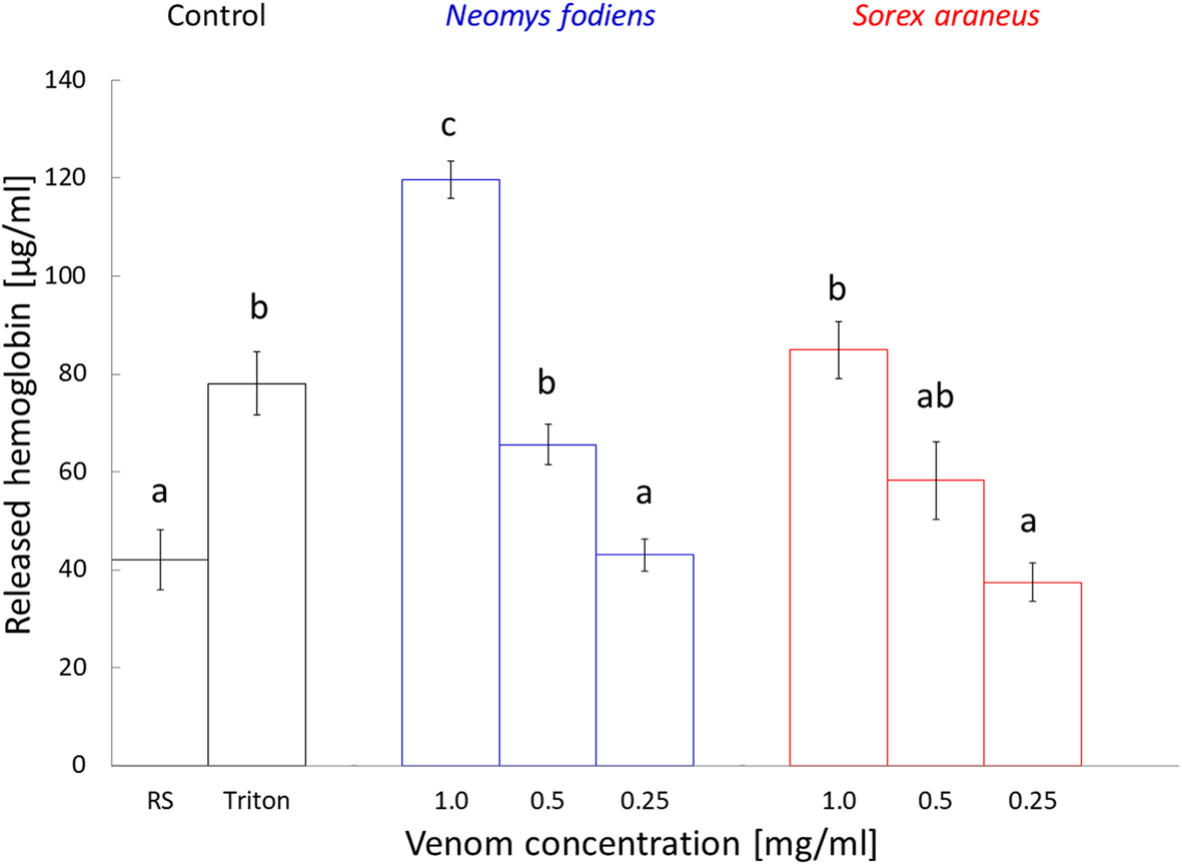
Hemolysis produced by shrew venoms expressed as concentration of released hemoglobin. Bars represent mean ± SEM. Different letters represent significant differences between treatments, controls and species

According to our predictions, Triton® X-100 (positive control) added to the red blood cells of frogs caused hemolysis when compared to the treatment with Ringer’s solution (RS; negative control; non-parametric Mann–Whitney U-test: U = 9.0, *p* < 0.005; Fig. 2).

The venom of *N. fodiens* added to the erythrocytes resulted in a concentration-dependent hemolytic response (non-parametric Kruskal–Wallis test: χ^2^ = 47.5, df = 2, *p* < 0.0001; Fig. 2), expressed as μg of released hemoglobin per ml. Treatment of erythrocytes with the venom at concentration of 1.0 mg/ml produced a significant increased release of hemoglobin as compared with control treatments (Fig. 2): RS (parametric Student’s t-test: t = -10.7, df = 28, *p* < 0.0001) and Triton (U = 18, *p* < 0.0001). At concentration of 0.5 mg/ml, it caused a higher hemoglobin release as compared with RS (t = -3.2, df = 28, *p* < 0.01), but similar to that caused by Triton (U = 151, *p* = 0.17). Venom of *N. fodiens* at concentration of 0.25 mg/ml did not cause significant changes in hemoglobin concentration as compared with RS (U = 86.5, *p* = 0.72), but the concentration of released hemoglobin was lower in comparison to the treatment with Triton (U = 209, *p* < 0.0001).

Venom of *N. fodiens* at concentration of 1.0 mg/ml added to erythrocytes produced a higher increase in hemoglobin concentration as compared with the treatments with venom concentrations of 0.5 and 0.25 mg/ml (Fig. 2; t = 9.6, df = 40, *p* < 0.0001, and U = 441, *p* < 0.0001, respectively). Also, addition of venom at concentration of 0.5 mg/ml caused a higher hemoglobin release in comparison to the treatment with venom concentration of 0.25 mg/ml (U = 370.5, *p* < 0.001).

Addition of the venom of *S. araneus* to the red blood cells also resulted in a concentration-dependent hemolytic response (*χ*^2^ = 31.7, df = 2, *p* < 0.0001; Fig. 2). Treatment with venom at concentration of 1.0 mg/ml caused a significant increased release of hemoglobin when compared with RS (U = 2, *p* < 0.001), but it was similar to the treatment with Triton (U = 27, *p* = 0.33). At concentration of 0.5 mg/ml, it did not produce changes in hemoglobin concentration as compared with RS (U = 17, *p* = 0.13) and Triton (U = 59, *p* = 0.07). Similarly, addition of *S. araneus* venom at concentration of 0.25 mg/ml to erythrocytes did not cause any changes in the concentration of released hemoglobin when compared with RS (U = 47, *p* = 0.90), but the hemoglobin concentration was lower when compared to the treatment with Triton (U = 110, *p* < 0.0001).

Treatment of the red blood cells with *S. araneus* venom at concentration of 1.0 mg/ml revealed a higher increase in the concentration of released hemoglobin in comparison to the treatments with venom at concentrations of 0.5 and 0.25 mg/ml (Fig. 2; U = 41, *p* < 0.05, and U = 70, *p* < 0.001, respectively). There were no differences in concentrations of released hemoglobin when treatments with venom at concentrations of 0.5 and 0.25 mg/ml were compared (U = 52, *p* = 0.11).

### Venom of *N. fodiens* has stronger hemolytic activity than venom of *S. araneus*

We found significant differences in hemolytic activity of venoms of *N. fodiens* and *S. araneus* (*χ*^2^ = 63.4, df = 5, *p* < 0.0001). *N. fodiens* venom at concentration of 1.0 mg/ml added to erythrocytes caused a higher increase in hemoglobin release than treatment with *S. araneus* venom at the same concentration (Fig. 2; U = 141, *p* < 0.0001). There were no differences in concentrations of released hemoglobin when treatments with *N. fodiens* and *S. araneus* venoms at concentration of 0.5 mg/ml were compared (U = 88, *p* = 0.47). The same was true for comparison of addition of *N. fodiens* and *S. araneus* venoms at concentrations of 0.25 mg/ml (U = 121, *p* = 0.52).

Venom of *N. fodiens* at concentration of 1.0 mg/ml caused a higher increase in hemoglobin release than *S. araneus* venom at concentrations of 0.5 and 0.25 mg/ml (U = 147, *p* < 0.0001, and U = 210, *p* < 0.0001, respectively). Also, addition of *N. fodiens* venom at concentration of 0.5 mg/ml caused a higher hemoglobin release in comparison to the treatment with *S. araneus* venom concentration of 0.25 mg/ml (U = 189, *p* = 0.0001).

Venom of *S. araneus* at concentration of 1.0 mg/ml caused a higher increase in hemoglobin release than *N. fodiens* venom at concentrations of 0.5 and 0.25 mg/ml (U = 112, *p* = 0.04, and U = 142, *p* < 0.0001, respectively). There were no differences in concentrations of released hemoglobin when treatments with *S. araneus* venom at concentration of 0.5 mg/ml and *N. fodiens* venom at concentration of 0.25 mg/ml were compared (U = 106, *p* = 0.09).

### Toxins identified in shrew venoms

**Fig. 3:**
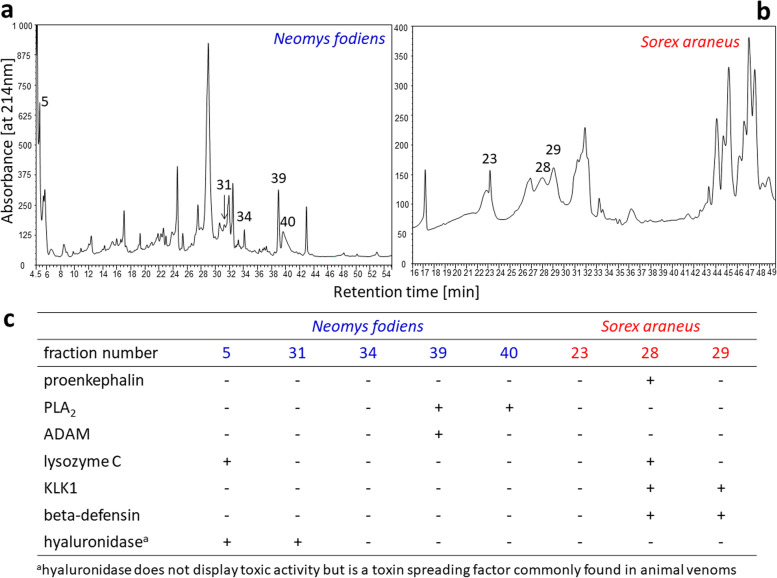
Chromatographic separation of the extract from venom glands of *Neomys fodiens* (**a**) and *Sorex araneus* (**b**). Numbers indicate the fractions selected to identify toxins. (**c**) Toxins identified in the selected fractions (see Additional files 1 and 2 in the Supplementary information section for the complete list of proteins identified in the extracts from venom glands of both shrew species). Abbreviations: ADAM, a disintegrin and metalloproteinase domain-containing protein; KLK1, kallikrein 1-related peptidase; PLA_2_, phospholipase A_2_. ( +) indicates the presence while (-) indicates the absence of toxin in the given fraction

We analyzed protein content in both the whole extract from venom glands of *N. fodiens* and *S. araneus* and in fractions separated by high-performance liquid chromatography (Fig. 3a,b) using MS/MS based proteomic techniques. We identified four toxins: proenkephalin-A, phospholipase A_2_ (PLA_2_), a disintegrin and metalloproteinase domain-containing protein (ADAM) and lysozyme C (Table 1; Fig. 3c) in the extract from venom glands of *N. fodiens*. We also found a hyaluronidase, a toxin spreading factor, in *N. fodiens* venom. We found five toxins, namely proenkephalin-A, kallikrein 1-related peptidase, beta-defensin 7, ADAM and lysozyme C (Table 1; Fig. 3c) in the extract from venom glands of *S. araneus*.

**Table 1:** Toxins identified in venoms of the Eurasian water shrew, *Neomys fodiens*, and the common shrew, *Sorex araneus*

Shrew species	Accession code	Identified peptides	Protein name
***Neomys fodiens***	P01211	K.LPSLKTWETCK.E K.KYGGFMK.R K.YGGFMK.R K.RYGGFLK.R	Proenkephalin-A
	P14422	FAKFLSYK	Phospholipase A_2_
	Q9Z0F8	SEDIKDFSR	Disintegrin and metalloproteinase domain-containing protein 17
	P12067	YWCNDGK	Lysozyme C
	gi|521,028,001	KDIEFYIPK	Hyaluronidase PH-20^a^
***Sorex araneus***	P01211	YGGFMK + Oxidation (M)	Proenkephalin-A
	Q61754	DKSNDLMLLR	Kallikrein 1-related peptidase b24
	Q91V70	FQIPEK	Beta-defensin 7
	Q10741	LYSDGKK	Disintegrin and metalloproteinase domain-containing protein 10
	P12067	AWVAWR	Lysozyme C

^a^hyaluronidase does not display toxic activity but is a toxin spreading factor commonly found in animal venoms

### Non-toxic molecules identified in shrew venoms

We identified 194 proteins in *N. fodiens* venom gland extract involved in various aspects of cell functioning such as: cell maintenance, cell cycle regulation, cell division, cell migration, cell adhesion and cell–cell interactions, cell aging and apoptosis, intra- and extracellular transport, signal transduction, energy metabolism, lipid metabolism, immune and stress response, and blood coagulation.

We found 112 proteins in the venom gland extract of *S. araneus* involved in cell maintenance, cell cycle regulation and cell division, cell migration and cell–cell interactions, cell respiration and apoptosis, intra- and extracellular transport, signal transduction, energy metabolism, lipid metabolism, immune and stress response, and blood coagulation. All proteins determined in the extracts from venom glands of *N. fodiens* and *S. araneus* and in separated fractions are listed in Supplementary Tables A1 and A2, respectively (see Additional files 1 and 2).

## Discussion

Shrew venoms are known to display strong paralytic and hypotensive in vitro and in vivo effects, which may enable hunting and storing prey in a comatose state [7, 9, 12, 13, 15]. Our study provides the first experimental evidence that shrew venoms produce potent hemolysis in the red blood cells of frogs, allowing shrews to hunt and kill larger prey such as amphibians. Intriguingly, for the first time, our results confirm the toxicity of saliva of *S. araneus*, which enables us to classify the common shrew as a venomous mammal. These findings have important implications for understanding the ecological functions and evolution of venoms in eulipotyphlans.

Venoms of many animal taxa, including snakes and lizards, fish, wasp and ants, jellyfish and anemones – but not mammals – have been proven to produce hemolytic effects [36–43]. For instance, venoms of Australian elapid snakes *Austrelaps superbus* and *Pseudechis colletti* produce high direct hemolysis of washed rabbit erythrocytes due to the hydrolysis of phosphatidylcholine in the cell membrane to fatty acids and glycerophosphorylcholine by phospholipase B [23]. Fernández et al. [39] demonstrated strong hemolytic effects induced by the venom of the Eastern coral snake, *Micrurus fulvius*, after intravenous injection into mice and dogs. Similarly, Lenske et al. [41] observed severe hemolysis in a dog envenomed by a red-bellied black snake, *Pseudechis porphyriacus*, venom. Also, venoms of the Gila monsters (genus *Heloderma*) *H. suspectum* and *H. horridum* are known to produce hemolysis [37]. Gilatoxins present in their saliva are devoid of PLA_2_ activity, but display proteolytic, hemorrhagic and hemolytic properties [37].

Cytolysin, a cytolytic toxin isolated from *Scorpaena plumieri* scorpionfish venom, showed a potent hemolytic activity on washed rabbit erythrocytes [38]. Venom of the Central American scorpion *Didymocentrus krausi* leads to direct hemolysis on human and rabbit erythrocytes [30]. Also, hemolysin isolated from *Pogonomyrmex badius* ant venom causes direct hemolysis on bovine and mouse washed erythrocytes [36, 44].

Our results confirmed, for both shrew species, significant concentration-dependent effects of venom on hemolysis of the red blood cells of frogs. Similar concentration-dependent hemolytic effects have recently been found in cnidarian venoms [40, 43]. It is noteworthy that most studies on hemolytic action of animal venoms have been performed on laboratory animals (including mice, rabbits and dogs) or humans. In our study, we used *Pelophylax* sp. frogs, which in nature are hunted by both *N. fodiens* and *S. araneus* [16, 33, 45, 46]. Therefore, we conclude that venom may help shrews to subdue and consume larger prey such as frogs.

Our results also show that hemolytic effects of *N. fodiens* venom are stronger than those produced by *S. araneus* venom. Both field observations and laboratory experiments indicate that *N. fodiens* is more effective in hunting and consuming frogs than *S. araneus* [33, 47]. This might be because *N. fodiens* produces stronger venom than *S. araneus* does, as demonstrated in this study. Thus, shrew venoms might have evolved not only to paralyze and immobilize prey to make food stores but also to kill and eat it quickly to meet their high energetic demands [7]. These findings are useful for understanding ecological functions of venom and predator–prey interactions.

Animal venoms are extremely rich and complex sources of biologically active molecules (usually proteins) with diverse toxicity [20–22] that are responsible for the pathophysiological consequences of envenoming [20]. Venom of a single animal may contain more than 1000 toxins [22, 48] making the venom a potent tool in hunting prey and repelling predators and competitors [2, 22]. Intriguingly, despite the varied diets of shrews [33, 47, 49, 50], toxin diversity in shrew venoms appears to be relatively low suggesting that the use of venom by shrews may be of recent evolutionary origin [7, 15].

Venom of *B. brevicauda* consists of six toxins, including blarina toxin (BLTX), soricidin, kallikrein-1 serine protease paralog (KLK1-BL2), PLA_2_, antileukoproteinase, and a tissue factor pathway inhibitor 2 protein, as well as four non-toxic proteins: hyaluronidase, blarinasin, and two KLK1 paralogs [12, 15, 19, 51]. The venom of *N. fodiens* also appears to have a relatively simple composition [13]. In the present study, we identified 199 proteins (see Additional file 1), including four toxins (Table 1), in the extract from venom glands of *N. fodiens*, and 117 proteins (see Additional file 2), including five toxins (Table 1), in the extract from venom glands of *S. araneus*. Thus, our results also confirm the simple composition of shrew venoms. Admittedly, it seems still possible to identify new molecules with toxic activity in shrew venoms, but it is not likely that their content would reach several hundred toxins as in venoms of *Conus* snails [48].

Lysozyme C, PLA_2_ and hyaluronidase identified here in *N. fodiens* venom have been previously reported in this species [3, 13]. Similarly, lysozyme C, KLK1 and beta-defensin have been found in the saliva of *S. araneus* [13]. Proenkephalin and a disintegrin and metalloproteinase domain-containing protein (ADAM) are reported here for both shrew species for the first time. Proenkephalin, which contains the known toxin peptide soricidin, has recently been characterized in the venom of *B. brevicauda* [15]. Therefore, identification of this molecule in venoms of *N. fodiens* and *S. araneus* suggests that soricidin may be more common among venomous shrews.

Metalloproteinases (ADAMs) have not been found in shrew venoms thus far, but they commonly occur in venoms of other taxa, including snakes, scorpions and jellyfish [24, 29, 30, 32, 52]. These molecules display hemolytic activity [32, 52]; therefore it is possible that hemolysis produced by shrew venoms in frog erythrocytes may be caused, at least partially, by ADAMs.

PLA_2_, here found only in the venom of *N. fodiens*, is widely distributed among elapid and viperid snake venoms and in scorpion venoms [27, 53, 54], and is also known for its cytotoxicity, including hemolytic activity [27, 29, 40, 55, 56]. For instance, PLA_2_ from *Cerastes ceratses* venom produces indirect hemolysis [56]. Lazcano-Pérez et al. [40] confirmed a dose-dependent hemolytic activity of cnidarian venom, which contains PLA_2_. Hemolytic effects of PLA_2_ from marine snail and wasp venoms have been also demonstrated [25, 42]. Thus, we consider PLA_2_ to be the most likely candidate toxin that could be responsible for the hemolytic properties of venom of *N. fodiens*. However, further studies with isolated venom components are required to confirm whether PLA_2_ or ADAM contained in shrew venoms produces hemolytic effects. The mode of action of these molecules also needs further validation.

Because lysozyme C is involved in antimicrobial defense [57, 58], it was thought to help in the maintenance of oral hygiene in venomous mammals [7]. Although there are some components with antimicrobial activity in venoms of *B. brevicauda* and *N. fodiens* [13, 15], this venom function has never been empirically tested in shrews [7]. Defensins, here identified in the venom of *S. araneus*, have previously been found in platypus venom [59]. These molecules exhibit a myo- and neurotoxic activity, modifying voltage-sensitive sodium channels, which usually results in a potent analgesic effect [60].

Kallikrein-like proteins, here also found in the venom of *S. araneus*, have earlier been detected in the venom of *B. brevicauda* [12]. BLTX has a tissue kallikrein-like protease activity. This toxin cleaves kininogens to kinins, such as bradykinin, a common mediator of inflammation that increases vascular permeability and lowers blood pressure [12]. Other kallikrein-1 paralogs in *B. brevicauda* venom have recently been discovered by Hanf and Chavez [15]. Therefore, these kinins are thought to be the primary toxic agents of shrew venom responsible for symptoms such as dyspnea, hypotension and hypokinesia, recorded previously in pharmacological studies [12].

As in our previous study [13], here we found a hyaluronidase in the venom of *N. fodiens*. This non-toxic protein is commonly present in many animal venoms, including venoms of snakes, spiders and ants [44, 61, 62], and acts as a toxin spreading factor [60, 61]. Due to its ability to hydrolyze connective tissue, hyaluronidase facilitates the action of other venom components [26]. Thus, it is possible that also in the venom of *N. fodiens* hyaluronidase facilitates the spread of venom proteins [13]. However, how the toxins are spread still requires investigation.

We provide here, for the first time, a comprehensive list of non-toxic proteins contained in venoms of both shrew species. Similarly, numerous non-toxic components have recently been identified in the venom and saliva of the Hispaniolan solenodon, *Solenodon paradoxus* [14]. These proteins may be responsible for the maintenance of homeostasis, synthesis of complex toxins, and their secretion into the extracellular compartment and secretion upregulation. We also found many proteins related to cell division and cell cycle regulation, which might indicate a high epithelial cell turnover in the venom glands of shrews.

The high number of non-toxic components found in our study may result from analyzing the whole extracts from salivary glands of shrews instead of crude venom, of which only limited amounts can be extracted in shrews [7]. As animals usually inject only venom into a target species during a bite, we recommend extracting only venom for future biochemical studies. On the other hand, to understand the molecular mechanisms underlying the evolution of shrew venoms it is necessary to study not only the venom toxins but also the molecular machinery related to venom production and secretion.

Proteins recruited into venoms and acting as toxins might also be present in non-venomous animals, but devoid of toxic activity [20, 21]. None of the venom proteins directly identified here show similarity to toxins reported in other venomous mammals. Therefore, in future studies we suggest using genomic, transcriptomic and proteomic data to predict venom protein identity based on sequence similarity to previously described animal toxins. Finally, to validate the toxicological role of venom proteins, it is also necessary to ascertain their mode of action.

Venom has evolved multiple times throughout the animal kingdom, but is rare among mammals [5, 63, 64]. However, it must be emphasized that most of potentially venomous mammal species have not yet been studied [7]. One of the first suggestions that shrews are venomous is from a description of the European common shrew, *Sorex araneus*, in *Historie of Foure-footed Beasts*: “It is a ravening beast, feigning itself gentle and tame, but being touched it biteth deep, and poisoneth deadly. It beareth a cruel mind, desiring to hurt anything, neither is there any creature it loveth” [65]. Even the Latin name (*aranea*) suggests that it would have venomous bites like a spider. However, shrew venoms are not harmful or deadly to humans. According to our personal experience, a bite of a water shrew on a finger causes at most some pain, swelling or numbness of the finger for 1–2 days.

Recent studies confirmed that *B. brevicauda* and *N. fodiens* produce toxic substances in their salivary glands [12, 13, 15], but toxic effects of the saliva of *S. araneus* were not confirmed [13]. Here, our study provides the first experimental evidence that saliva of the common shrew has cytotoxic activity leading to hemolysis in the red blood cells of frog. Thus, our results prove, confirming a suggestion made four centuries ago [65], that *S. araneus* is indeed venomous. This is also the first report confirming venom production in a member of the genus *Sorex*, which includes 86 shrew species [66]. This suggests that more venomous shrew species may be discovered in the future. Two more *Sorex* species – *S. palustris* and *S. cinereus* – are already suspected of being venomous [4, 7], but the toxicity of their saliva has not yet been studied. Further research will allow us to investigate questions about how mammalian venoms have evolved. It is likely that venom production in shrews, and other eulipotyphlans, may be more widespread than it has previously been assumed. However, much more research effort is required to determine which shrew species do or do not produce venom and how widespread venom truly is. Finally, because animal toxins have applications in medicine and pharmacy [19, 67, 68], characterization of new toxins in eulipotyphlan venoms, and in so numerous and widespread species as *S. araneus*, provides a promising avenue to explore bioactive venom components as therapeutic agents in the future [69, 70].

## Conclusions

Our results clearly show that shrew venoms produce hemolysis in frog erythrocytes, which suggests that this may allow these shrews to hunt larger prey. Because the venom of *N. fodiens* causes stronger hemolytic effects than venom of *S. araneus*, *N. fodiens* may be more effective in killing and storing such large prey as frogs than *S. araneus*. Since a member of the numerous genus *Sorex* is venomous, it is likely that venom production among shrews and other eulipotyphlans may be more widespread than it has previously been assumed.

## Materials and methods

### Animals

#### Shrews

Trapping sessions were performed in the suburbs of Poznań (western Poland) from July to September 2017. In total, we captured 12 water shrews and 12 common shrews. The captured animals were transported to the laboratory and placed separately into large (39 × 21 × 28 cm; 23 l) terraria equipped with bedding (a mixture of peat, moss and sand). Each terrarium contained a shelter (upturned clay flowerpot) and a bowl of water. Food (minced beef and live mealworms, earthworms and snails) and water were provided *ad libitum.* Shrews were kept in an animal room under controlled conditions (temperature: 21 ± 1 °C; humidity: 65–70%; artificial photoperiod: 12L:12D). After a week, they were killed using approved methods to obtain their submandibular salivary glands, in which toxins are produced [13].

#### Frogs

Nine frogs of the genus of *Pelophylax* sp. were captured using a net near ponds and small water tanks located in the Morasko district of Poznań (western Poland). Captured animals were placed into plastic transporters and carried to the laboratory, where their body mass (mean m_b_ [g] ± SEM: 126.2 ± 12.8) and snout to vent length (mean SVL [mm] ± SEM: 92.4 ± 3.84) were measured. Next, they were placed into large (46 × 30 × 28 cm; 39 l) aqua-terraria (up to 3 animals per terrarium) equipped with bedding (a mixture of peat and sand). The terraria were regularly irrigated to maintain high humidity. Each terrarium contained a shelter (clay flowerpot) and a water tank to allow frogs to submerge in water. Food (live mealworms and crickets) and water were provided *ad libitum.* Frogs were kept (until the experimental procedure began) in the animal room under controlled conditions (temperature: 20 ± 1 °C; humidity: 65–70%; artificial photoperiod: 12L:12D).

### Venom collection and sample preparation

Shrews were killed by cervical vertebrae dislocation, and their submandibular salivary (venom) glands were dissected to obtain toxic saliva [13]. Pairs of glands (*n* = 2; from each shrew species) designed for hemolytic bioassays were transferred into 600 μl of Ringer’s solution (RS) for frogs (125 mM NaCl; 3 mM KCl; 1.8 mM CaCl_2_; 10 mM glucose; 5 mM HEPES). Pairs of glands (*n* = 10, from each shrew species) designed for chromatographic separation were transferred into 600 μl of methanol. Tissues were next homogenized, and samples were centrifuged at 10,000 × g and 4 °C for 30 min. The supernatants were collected, and the protein content was determined using a Direct Detect spectrometer (MERCK Millipore, Warsaw, Poland).

### Chromatographic separation

Supernatants suspended in methanol were used for separation before peptide analysis by reverse phase high-performance liquid chromatography (RP-HPLC). Separation was performed using a Dionex Ultimate 3000 chromatographic system comprising a dual pump programmable solvent module. Supernatants were analyzed using a BioBasic-18 analytical column (5 μm, 150 × 4.6 mm; Thermo Fisher Scientific). The samples were eluted with a gradient of 5–60% acetonitrile (ACN)/0.1% TFA with a flow rate of 0.5 ml/min for 55 min. The eluent was monitored at 214 nm, and fractions were collected into 1.5-ml tubes.

### Hemolytic assay

Frogs were killed using approved methods [13] and blood was sampled from the heart and put into a tube containing 5 μl of 3.2% sodium citrate, an anticoagulant. Two blood samples were additionally taken using heparinized capillary tubes to estimate the hematocrit (mean Ht [%] ± SEM: 28.3 ± 0.77). Blood (50 μl) was re-suspended in 50 μl of RS and centrifuged at 1500 × g for 5 min at 4 °C. The supernatants were then removed, 100 μl of RS was added and the procedure was repeated two more times. The pellets were finally diluted with 100 μl of RS. Next, 10- and 100-fold dilutions were prepared, and were used for the experiments. The erythrocytes were then incubated (final volume 100 μl) at 25 °C for 1 h in the presence of *N. fodiens* saliva (with protein concentrations of 1.0, 0.5, and 0.25 mg/ml) or *S. araneus* saliva (with the same protein concentrations), and centrifuged at 1500 × g for 5 min at 4 °C. Samples containing red blood cells with RS were used as a negative control, whereas samples with Triton® X-100 as a positive control. The number of replicates (n) for each venom concentration was as follows: 21 for *N. fodiens*, 7 to 10 for *S. araneus*, 9 for RS and 11 for Triton. The released hemoglobin was measured in the final supernatants using a spectrophotometer at 415 nm, and compared with a standard curve constructed with bovine hemoglobin [71]. The results were expressed as μg hemoglobin per ml.

### Protein and toxin identification

Proteomic analysis was carried out at the Mass Spectrometry Laboratory, Institute of Biochemistry and Biophysics, Polish Academy of Sciences, Warsaw. Peptides from the whole extract and separated fractions (that proved to be the most active [13] and with the highest protein content; Fig. 1d,e) of *N. fodiens* and *S. araneus* venom glands were analyzed by liquid chromatography coupled to tandem mass spectrometry LC-(MS–MS/MS) using a Nano-Acquity LC system (Waters, Milford, MA) and an OrbitrapVelos mass spectrometer (Thermo Electron Corp., San Jose, CA).

Before performing the analysis, the proteins were subjected to an ion-solution digestion procedure. Proteins were (1) reduced with 50 mM TCEP for 30 min at 60 °C, (2) alkylated with 200 mM MMTA for 30 min at room temperature and (3) digested overnight with trypsin (sequencing Grade Modified Trypsin—Promega V5111). Next, the samples were applied to an RP-18 precolumn (nanoACQUITY Symmetry® C18—Waters 186,003,514) using water containing 0.1% TFA as a mobile phase and were transferred to a nano-HPLC RP-18 column (nanoACQUITY BEH C18 – Waters 186,003,545). The samples were eluted with a gradient of 0–35% acetonitrile in the presence of 0.05% formic acid with a flow rate of 250 nl/min for 180 min. The column was directly coupled to the ion source of the spectrometer working within data dependent on the MS to MS/MS switch. To ensure a lack of cross contamination from previous samples, each analysis was preceded by a blank run.

The proteins were identified by a Mascot Search (Matrix Science, London, UK) against the SwissProt and NCBInr databases. Because only a few toxins have been identified in shrew venoms so far, and only in two shrew species, we also searched for toxin/protein sequences found in other venomous animal taxa. The search parameters were as follows: type of search: MS/MS Ion Search; enzyme specificity: trypsin; fixed methylthio modification of cysteine; variable modifications: methionine oxidation; mass values: monoisotopic; protein mass: unrestricted; peptide mass tolerance: 30 ppm; fragment mass tolerance: 0.1 D; number of missed cleavage sites allowed: 1; instrument type: HCD. Peptides with Mascot scores exceeding the threshold value of *p* < 0.05 were considered positively identified.

### Data analysis

All data are presented as the mean value ± SEM (standard error of the mean) of the indicated number of replicates (n). Before the statistical analysis, the normality of the data distribution was checked using the Shapiro–Wilk test. To determine the effects of saliva of both shrew species on hemolysis in frog erythrocytes the Kruskal–Wallis test was used. The mean differences between the treatment (number of replicates for each venom concentration: *n* = 21 for *N. fodiens* and *n* = 7–10 for *S. araneus*) and control groups (*n* = 9 for RS and *n* = 11 for Triton) were determined using the paired two-sided Student’s t-test or Wilcoxon signed-rank test. To indicate statistically significant differences between hemolytic activity of *N. fodiens* saliva and *S. araneus* saliva, the Mann–Whitney U-test was performed. Non-parametric tests (Wilcoxon signed-rank and Mann–Whitney) were used when the datasets of non-normal distributions were compared. The statistical analyses were carried out using R software [72]. Significant results were considered those with a *p*-value of *p* < 0.05.

## Supplementary Information

**Additional file 1: Table A1.** Protein identification in the extract from venom glands of the Eurasian water shrew, *Neomys fodiens*, based on tandem mass spectrometry analysis. Toxins are shown in bold.

**Additional file 2: Table A2.** Protein identification in the extract from venom glands of the common shrew, *Sorex araneus*, based on tandem mass spectrometry analysis. Toxins are shown in bold.

## Data Availability

All data are available in the main text and the supplementary information files. Further information and requests for data should be directed to and will be fulfilled by the corresponding author.

## Abbreviations

ADAM: A disintegrin and metalloproteinase domain-containing protein
ADAMs: Metalloproteinases
BLTX: Blarina Toxin
Ht: Hematocrit
KLK1-BL2: Kallikrein-1 serine protease paralog
LC-(MS–MS/MS): Liquid Chromatography Coupled to Tandem Mass Spectrometry
m_b_: Body mass
PLA_2_: Phospholipase A_2_
RP-HPLC: Reverse Phase High-performance Liquid Chromatography
RS: Ringer’s Solution
SVL: Snout to Vent Length
SVMPs: Snake Venom Metalloproteinases
