# Supplementary material for: A new, widespread venomous mammal species: hemolytic activity of Sorex araneus venom is similar to that of Neomys fodiens venom
Source: Zoological Lett. 2022 Jun 7;8:7. doi: 10.1186/s40851-022-00191-5 (PMC9172195; doi:10.1186/s40851-022-00191-5)
Supplement: Supplementary file 1 — Additional file 1: Table A1. Protein identification in the extract from venom glands of the Eurasian water shrew, Neomys fodiens, based on tandem mass spectrometry analysis. Toxins are shown in bold. [file 40851_2022_191_MOESM1_ESM.docx]

**A new, widespread venomous mammal species: hemolytic activity of *Sorex araneus* venom is similar to that of *Neomys fodiens* venom**

Krzysztof Kowalski^1*^, Paweł Marciniak^2^ and Leszek Rychlik^3^

^1^Department of Vertebrate Zoology and Ecology, Institute of Biology, Faculty of Biological and Veterinary Sciences, Nicolaus Copernicus University, Lwowska 1, Toruń, 87-100, Poland, e-mail: k.kowalski@umk.pl,: tel. +48 56 611 4910

^2^Department of Animal Physiology and Developmental Biology, Institute of Experimental Biology, Faculty of Biology, Adam Mickiewicz University, Uniwersytetu Poznańskiego 6, Poznań, 61-614, Poland, e-mail: pmarcin@amu.edu.pl, tel.: +48 61 829 5926

^3^Department of Systematic Zoology, Institute of Environmental Biology, Faculty of Biology, Adam Mickiewicz University, Uniwersytetu Poznańskiego 6, Poznań, 61-614, Poland, e-mail: leszek.rychlik@amu.edu.pl, tel.: +48 61 829 5751

*Correspondence: k.kowalski@umk.pl (K.K.)

**Table A1** Protein identification in the extract from venom glands of the Eurasian water shrew *Neomys fodiens* based on tandem mass spectrometry analysis. Toxins are shown in bold.

| **Accession** | **Matched peptides** | **Protein sequence coverage [%]** | **Ion score** | **m/z** | **Identified peptides** | **Protein name** | **Species** |
| --- | --- | --- | --- | --- | --- | --- | --- |
| **whole extract** | | | | | | | |
| Q9D6P8 | 25 | 37 | 64  37  57  97  81  65 | 955  4086  4102  1351  1367  1092 | K.EAFSLFDK.D  R.SLGQNPTEAELQGMVNEIDKDGNGTVDFPEFLTMMSR.K + Oxidation (M)  R.SLGQNPTEAELQGMVNEIDKDGNGTVDFPEFLTMMSR.K + 2 Oxidation (M)  K.MKDTDSEEEIR.E  K.MKDTDSEEEIR.E + Oxidation (M)  K.DTDSEEEIR.E | Calmodulin-like  protein 3 | *Mus musculus* |
| Q3T140 | 49 | 51 | 101  100  79  80 | 1636  2439  2455  1460 | K.GVQGIIVVNTEGIPIK.S  K.STMDNPTTTQYANLMHNFILK.A  K.STMDNPTTTQYANLMHNFILK.A + Oxidation (M)  R.EIDPQNDLTFLR.I | Dynein light chain roadblock-type 1 | *Bos taurus* |
| Q6P7Q4 | 64 | 33 | 78  67  60  48  76  49  114 | 1264  1280  1028  900  1962  976  2288 | K.DFLLQQTMLR.I  K.DFLLQQTMLR.I + Oxidation (M)  K.KSLDFYTR.V  K.SLDFYTR.V  K.FSLYFLAYEDKNDIPK.D  K.RFEELGVK.F  K.GLAFVQDPDGYWIEILNPNK.M | Lactoylglutathione lyase | *Rattus norvegicus* |
| Q2KJG2 | 54 | 68 | 54  75  59  63  108 | 901  1587  840  3575  2752 | K.ITLTSDPR.L  K.VLSVPESTPFTAVLK.F  K.FAAEEFK.V  K.FAAEEFKVPAATSAIITNDGIGINPAQTAGNVFLK.H  K.VPAATSAIITNDGIGINPAQTAGNVFLK.H | Ubiquitin-fold modifier 1 | *Bos taurus* |
| P31044 | 35 | 6 | 75  59  96  90 | 1742  1758  1439  1455 | K.FREWHHFLVVNMK.G  K.FREWHHFLVVNMK.G + Oxidation (M)  R.EWHHFLVVNMK.G  R.EWHHFLVVNMK.G + Oxidation (M) | Phosphatidylethanolamine-binding protein 1 | *Rattus norvegicus* |
| A4Z6H1 | 28 | 16 | 105  140  84  79  52 | 2209  2286  1444  1302  1330 | R.AHIVFDFHQAADGIQEQQR.Q  R.VGIGAFPTEQDNEIGELLQTR.G  K.TLPGWNTDISNAR.T  K.ELPVNAQNYVR.F  R.FIEDELQIPVK.W | Adenylosuccinate synthetase isozyme 2 | *Sus scrofa* |
| Q3SZ62 | 28 | 21 | 87  98  49  78  75  55 | 1058  1683  1306  1150  2114  2242 | R.HYGGLTGLNK.A  R.ALPFWNEEIVPQIK.E  K.RVLIAAHGNSLR.G  R.VLIAAHGNSLR.G  K.NLKPIKPMQFLGDEETVR.K  K.NLKPIKPMQFLGDEETVRK.A | Phosphoglycerate mutase 1 | *Bos taurus* |
| O35586 | 24 | 8 | 107 | 2233 | K.AQSDGIWGEHEIDYILFLK.K | Isopentenyl-diphosphate Delta-isomerase 1 | *Mesocricetus auratus* |
| Q1ZZU7 | 15 | 15 | 107  99  45 | 1273  1289  714 | M.PMFVVNTNVPR.A  M.PMFVVNTNVPR.A + Oxidation (M)  K.IGGAQNR.S | Macrophage migration inhibitory factor | *Ovis aries* |
| Q45FY6 | 28 | 22 | 65  56  66  74  48 | 1297  1313  1178  1428  1825 | K.VFIPHGLIMDR.T  K.VFIPHGLIMDR.T + Oxidation (M)  R.SIPMTVDFIR.L  K.NVLIVEDIIDTGK.T  R.SVGYRPDFVGFEIPDK.F | Hypoxanthine-guanine phosphoribosyltransferase | *Sus scrofa* |
| O89106 | 16 | 30 | 63  47  107  75 | 1612  2365  2381  1093 | R.FGQHLIKPSVVFLK.T  K.HFQGTSITFSMQDGPEAGQTVK.H  K.HFQGTSITFSMQDGPEAGQTVK.H + Oxidation (M)  K.HVHVHVLPR.K | Bis(5’-adenosyl)-triphosphatase | *Mus musculus* |
| Q3T0F4 | 8 | 9 | 127  123 | 1569  1440 | K.KAEAGAGSATEFQFR.G  K.AEAGAGSATEFQFR.G | 40S ribosomal protein S10 | *Bos taurus* |
| P07107 | 23 | 22 | 62  54  46 | 1260  1060  1207 | K.AKWDAWNELK.G  K.WDAWNELK.G  K.AYIDKVEELK.K | Acyl-CoA-binding protein | *Bos taurus* |
| Q8WNN6 | 18 | 18 | 114  62  31  42 | 1167  842  858  989 | R.HVGDLGNVTAGK.D  R.TMVVHEK.R  R.TMVVHEK.R + Oxidation (M)  R.LACGVIGIAQ.- | Superoxide dismutase [Cu-Zn] | *Canis lupus familiaris* |
| O97680 | 19 | 28 | 71  59  44  55 | 1463  1479  1193  907 | K.MIKPFFHSLSEK.Y  K.MIKPFFHSLSEK.Y + Oxidation (M)  K.CMPTFQFFK.K  K.VGEFSGANK.E | Thioredoxin | *Bos taurus* |
| Q8K0C9 | 16 | 9 | 81  79  73  66 | 1028  1393  909  947 | K.GYEVHGIVR.R  K.FYQASTSELYGK.V  K.VHVTVDLK.Y  R.VAFDELVR.E | GDP-mannose 4,6 dehydratase | *Mus musculus* |
| P10462 | 6 | 16 | 128 | 1775 | K.ELPSFVGEKVDEEGLK.K | Protein S100-A2 | *Bos taurus* |
| Q9CR86 | 8 | 10 | 117 | 1675 | K.LQAVEVVITHLAPGTK.H | Calcium-regulated heat stable protein 1 | *Mus musculus* |
| B3EWE1 | 9 | 26 | 69  96 | 1793  2186 | K.TYFPHFDLSPGSAQVK.G  K.AVGSLDDLPGALSALSDLHAHK.L | Hemoglobin subunit alpha | *Blarina brevicauda* |
| P27213 | 8 | 15 | 81  102  89 | 1002  1456  1642 | R.LVSFSASHR.L  K.VYETDNNIVVYK.G  K.VYETDNNIVVYKGE.- | 6-pyruvoyl tetrahydrobiopterin synthase | *Rattus norvegicus* |
| Q2KIV2 | 5 | 16 | 109 | 1789 | R.FQEYHIQQNEALAAK.A | Mitochondrial import inner membrane translocase subunit Tim9 | *Bos taurus* |
| Q5S3G4 | 8 | 9 | 99 | 1328 | R.KGLDPYNILAPK.A | Cytochrome c oxidase subunit 5B, mitochondrial | *Sus scrofa* |
| Q60550 | 6 | 12 | 74  87 | 1351  1718 | M.PPYTIVYFPVR.G  K.EAALVDMANDGVEDLR.C | Glutathione S-transferase P | *Mesocricetus auratus* |
| P12815 | 6 | 12 | 64  79 | 1441  1356 | K.AGVNFSEFTGVWK.Y  R.LSDQFHDILIR.K | Programmed cell death protein 6 | *Mus musculus* |
| Q3T054 | 10 | 19 | 30  57  53  69 | 959  1293  1213  1784 | R.HLTGEFEK.K  K.FNVWDTAGQEK.F  K.NLQYYDISAK.S  K.SNYNFEKPFLWLAR.K | GTP-binding nuclear protein Ran | *Bos taurus* |
| Q2EN75 | 6 | 15 | 62  61 | 730  876 | K.ELTIGAK.L  K.LMDDLDR.N | Protein S100-A6 | *Sus scrofa* |
| P17563 | 6 | 10 | 83  39  62  27 | 1905  1050  1263  2546 | R.NTGTEAPDYLATVDVDPK.S  K.QFYPDLIR.E  K.LNPNFLVDFGK.E  K.LNPNFLVDFGKEPLGPALAHELR.Y | Selenium-binding protein 1 | *Mus musculus* |
| P01139 | 8 | 6 | 68  37 | 1153  765 | K.LQHSLDTALR.R  R.RLHSPR.V | Beta-nerve growth factor | *Mus musculus* |
| Q9D0J8 | 4 | 10 | 76 | 1074 | K.SVEAAAELSAK.D | Parathymosin | *Mus musculus* |
| P02049 | 3 | 8 | 81 | 1300 | K.VNVDDVGGEALGR.L | Hemoglobin subunit beta | *Nycticebus coucang* |
| B0VYY2 | 4 | 30 | 89  50 | 1632  3428 | K.GMNTLVGYDLVPEPK.I  K.EIYPYVIQELRPTLNELGISTPEELGLDKV.- | Cytochrome c oxidase subunit 5A, mitochondrial | *Nycticebus coucang* |
| P01867 | 4 | 8 | 71 | 4012 | K.VTCVVVDVSEDDPDVQISWFVNNVEVHTAQTQTHR.E | Ig gamma-2B chain C region | *Mus musculus* |
| P01289 | 3 | 8 | 71 | 1210 | K.ALYGHGQLSHK.R | Protachykinin-1 | *Bos taurus* |
| **P01211** | **6** | **9** | **27**  **37**  **49**  **28** | **1351**  **829**  **701**  **839** | **K.LPSLKTWETCK.E**  **K.KYGGFMK.R**  **K.YGGFMK.R**  **K.RYGGFLK.R** | **Proenkephalin-A** | ***Bos taurus*** |
| A2VE52 | 1 | 7 | 66 | 2178 | K.ESTMTLQQAEYEFLSFVR.Q | Oligoribonuclease, mitochondrial | *Bos taurus* |
| P21571 | 1 | 8 | 65 | 1061 | K.FEVLDKPQS.- | ATP synthase-coupling factor 6, mitochondrial | *Rattus norvegicus* |
| O97797 | 1 | 13 | 54 | 1584 | K.NSPFYYDWHSLR.V | FXYD domain-containing ion transport regulator 3 | *Sus scrofa* |
| P62077 | 2 | 13 | 43 | 1207 | R.FIDTTLAITGR.F | Mitochondrial import inner membrane translocase subunit Tim8 B | *Mus musculus* |
| Q3T0I5 | 1 | 7 | 45 | 1195 | K.GLALLEELLPK.G | Mitochondrial fission 1 protein | *Bos taurus* |
| Q3SZ68 | 1 | 28 | 44 | 1994 | R.GTLSDEHAGVISVLAQQAAK.L | Ragulator complex protein LAMTOR5 | *Bos taurus* |
| Q9CXP8 | 1 | 10 | 41 | 772 | K.LEAGVER.I | Guanine nucleotide-binding protein G(I)/G(S)/G(O) subunit gamma-10 | *Mus musculus* |
| Q3SZE2 | 1 | 20 | 40 | 2859 | K.KHAHLTDTEIMTLVDETNMYEGVGR.M | Prefoldin subunit 1 | *Bos taurus* |
| **P00592** | **2** | **4** | **37** | **819** | **R.ALWQFR.S** | **Phospholipase A2, major isoenzyme** | ***Sus scrofa*** |
| P43023 | 2 | 11 | 30 | 1120 | K.VLSRSMASAAK.G | Cytochrome c oxidase subunit 6A2, mitochondrial | *Mus musculus* |
| Q5SSZ7 | 13 | 17 | 29 | 859 | IQHLPPR | E3 ubiquitin-protein ligase ZNRF3 | *Mus musculus* |
| Q28103 | 2 | 12 | 28 | 2483 | R.IKERPALNAQDGIFVINPEMGR.S + Oxidation (M) | Microfibril-associated glycoprotein 3 (Fragment) | *Bos taurus* |
| P01637 | 12 | 11 | 28 | 841 | LVDGVPSR | Ig kappa chain V-V region T1 | *Mus musculus* |
| Q8VI38 | 9 | 10 | 27 | 906 | RMETINK + Oxidation (M) | Globoside alpha-1,3-N-acetylgalactosaminyltransferase 1 | *Mus musculus* |
| Q6DFX2 | 31 | 14 | 27 | 1355 | LANEQIQNAGGLK | Anthrax toxin receptor 2 | *Mus musculus* |
| Q9CX56 | 9 | 10 | 26 | 811 | TPRGEPR | 26S proteasome non-ATPase regulatory subunit 8 | *Mus musculus* |
| P21981 | 15 | 20 | 26 | 874 | QEDGSVLK | Protein-glutamine gamma-glutamyltransferase 2 | *Mus musculus* |
| Q8BV79 | 14 | 35 | 26 | 712 | LEQVPK | TPR and ankyrin repeat-containing protein 1 | *Mus musculus* |
| Q3SZP5 | 16 | 7 | 26 | 1515 | EVAWNLTSIDLVR | Peroxisomal acyl-coenzyme A oxidase 1 | *Bos taurus* |
| P46633 | 17 | 18 | 25 | 844 | EILQQSK | Heat shock protein HSP 90-alpha | *Cricetulus griseus* |
| Q17R09 | 27 | 10 | 25 | 1361 | MEDSGEDASLHR + Oxidation (M) | Pre-mRNA-splicing factor ATP-dependent RNA helicase PRP16 | *Bos taurus* |
| Q62924 | 16 | 18 | 25 | 822 | ELLFSSK | A-kinase anchor protein 11 | *Rattus norvegicus* |
| Q6PFY1 | 25 | 12 | 25 | 1485 | QQREADLLEDIR | F-BAR and double SH3 domains protein 1 | *Mus musculus* |
| Q8MKF1 | 12 | 17 | 25 | 903 | LIVYLQR | Thiamine-triphosphatase | *Bos taurus* |
| Q794H2 | 18 | 7 | 25 | 1613 | VEEEEAPKETPEVK | Nucleosome assembly protein 1-like 3 | *Mus musculus* |
| **Q8SQG8** | **14** | **8** | **24** | **980** | **HKMPLDPK** | **Hyaluronidase-2*** | ***Bos taurus*** |
| Q68FY1 | 20 | 8 | 24 | 1541 | GVLSSPSLAFTPPIR | Nucleoporin NUP53 | *Rattus norvegicus* |
| Q28141 | 9 | 8 | 24 | 973 | RLNMATLR | ATP-dependent RNA helicase A | *Bos taurus* |
| Q8BKF1 | 11 | 7 | 23 | 1156 | LQETLQSLPK | DNA-directed RNA polymerase, mitochondrial | *Mus musculus* |
| A1A5Q5 | 20 | 28 | 23 | 726 | RLPEGR | Lysine-specific demethylase 4D | *Rattus norvegicus* |
| Q5FVR0 | 13 | 13 | 23 | 760 | KNSGSLR | T-cell immunoglobulin and mucin domain-containing protein 2 | *Rattus norvegicus* |
| P35831 | 30 | 12 | 23 | 1329 | TSKPQELSAGALK | Tyrosine-protein phosphatase non-receptor type 12 | *Mus musculus* |
| Q8MIT6 | 11 | 21 | 23 | 771 | IGDLQAR | Rho-associated protein kinase 1 (Fragment) | *Bos taurus* |
| P21752 | 11 | 11 | 22 | 862 | KTETQEK | Thymosin β-10 | *Bos taurus* |
| Q3ZBM5 | 10 | 13 | 22 | 748 | ADRMTR | Sorting nexin-5 | *Bos taurus* |
| Q2KIE4 | 11 | 7 | 22 | 937 | MSAEDIEK + Oxidation (M) | Malignant T-cell-amplified sequence 1 | *Bos taurus* |
| Q9R103 | 30 | 14 | 22 | 1350 | SLNHSGETLHQK | Interleukin-12 subunit alpha | *Rattus norvegicus* |
| O08789 | 17 | 17 | 22 | 827 | LAPAEEAK | Max-binding protein MNT | *Mus musculus* |
| Q29466 | 17 | 10 | 22 | 1158 | EINTNQEALK | V-type proton ATPase 116 kDa subunit a isoform 1 | *Bos taurus* |
| Q1LZH0 | 6 | 10 | 22 | 877 | LKEVFSR | U11/U12 small nuclear ribonucleoprotein 35 kDa protein | *Bos taurus* |
| Q9JLI6 | 7 | 11 | 22 | 703 | FPGVER | Selenocysteine lyase | *Mus musculus* |
| P97807 | 17 | 10 | 22 | 1284 | KPVHPNDHVNK | Fumarate hydratase, mitochondrial | *Mus musculus* |
| Q3SZ22 | 12 | 8 | 21 | 1251 | FLGNAPCGHYK | 39S ribosomal protein L46, mitochondrial | *Bos taurus* |
| Q3UHX0 | 9 | 16 | 21 | 822 | YCHNIK | Nucleolar protein 8 | *Mus musculus* |
| Q2KIF8 | 17 | 18 | 21 | 775 | QDMAALK | Cysteine--tRNA ligase, mitochondrial | *Bos taurus* |
| Q99LC8 | 22 | 11 | 21 | 1414 | LFPLNQEDVPDK | Translation initiation factor eIF-2B subunit alpha | *Mus musculus* |
| **fraction 5** | | | | | | | |
| H6BDU4 | 4 | 7 | 82 | 1167 | R.HVGDLGNVTAGK.D | Superoxide dismutase [Cu-Zn] | *Camelus dromedarius* |
| P21571 | 3 | 8 | 54 | 1061 | K.FEVLDKPQS.- | ATP synthase-coupling factor 6, mitochondrial | *Rattus norvegicus* |
| B2RXB2 | 4 | 8 | 43 | 772 | R.IEDLQR.N | Heat shock factor-binding protein 1-like protein 1 | *Mus musculus* |
| P08814 | 1 | 11 | 34 | 1358 | R.AAEEEDEADPKR.Q | Parathymosin | *Bos taurus* |
| P14841 | 14 | 8 | 28 | 1207 | GTHTLTKSSCK | Cystatin-C | *Rattus norvegicus* |
| Q52RG8 | 12 | 12 | 27 | 852 | GWGTAHPK | Fibroblast growth factor receptor substrate 3 | *Rattus norvegicus* |
| Q5E9S2 | 16 | 10 | 26 | 1080 | GEGGRFFSPK | Nuclear transcription factor Y subunit alpha | *Bos taurus* |
| Q8BV79 | 11 | 16 | 26 | 712 | LEQVPK | TPR and ankyrin repeat-containing protein 1 | *Mus musculus* |
| Q921F4 | 15 | 11 | 26 | 852 | SSSSSSSPK | Heterogeneous nuclear ribonucleoprotein L-like | *Mus musculus* |
| Q8SPJ1 | 14 | 18 | 26 | 811 | LVQLLVK | Junction plakoglobin | *Bos taurus* |
| A6QNR1 | 16 | 12 | 25 | 1121 | MEQQEMAQK | Ribosomal RNA processing protein 36 homolog | *Bos taurus* |
| Q922J3 | 24 | 20 | 25 | 901 | QQLEGAEK | CAP-Gly domain-containing linker protein 1 | *Mus musculus* |
| Q99K01 | 15 | 21 | 25 | 738 | KVDHIK | Pyridoxal-dependent decarboxylase domain-containing protein 1 | *Mus musculus* |
| Q9EQH2 | 16 | 10 | 25 | 727 | EKPELL | Endoplasmic reticulum aminopeptidase 1 | *Mus musculus* |
| Q2TBI0 | 8 | 8 | 25 | 979 | HFGSVDYR | Lipopolysaccharide-binding protein | *Bos taurus* |
| **P12067** | **6** | **8** | **24** | **930** | **YWCNDGK** | **Lysozyme C-1** | ***Sus scrofa*** |
| P05008 | 9 | 8 | 24 | 817 | EGSSLAVR | Interferon alpha-B | *Bos taurus* |
| Q811I0 | 20 | 17 | 23 | 879 | KQPVGHSK | ATP synthase mitochondrial F1 complex assembly factor 1 | *Mus musculus* |
| Q7TNT2 | 19 | 19 | 23 | 841 | EDLAGIPK | Fatty acyl-CoA reductase 2 | *Mus musculus* |
| P26954 | 16 | 13 | 22 | 911 | EKIPNPSK | Interleukin-3 receptor class 2 subunit beta | *Mus musculus* |
| **Q8SQG8** | **7** | **4** | **22** | **980** | **HKMPLDPK + Oxidation (M)** | **Hyaluronidase-2*** | ***Bos taurus*** |
| O08789 | 17 | 17 | 22 | 827 | LAPAEEAK | Max-binding protein MNT | *Mus musculus* |
| O88855 | 6 | 10 | 22 | 752 | GTWSFR | Leukotriene B4 receptor 1 | *Mus musculus* |
| P01252 | 22 | 9 | 22 | 1566 | AAEDDEDDDVDTKK | Prothymosin alpha | *Bos taurus* |
| Q9EPQ8 | 16 | 9 | 22 | 1132 | ENDTVMISPK | Transcription factor 20 | *Mus musculus* |
| Q5I043 | 12 | 8 | 22 | 1091 | TLLEQFADR | Ubiquitin carboxyl-terminal hydrolase 28 | *Mus musculus* |
| P01637 | 12 | 11 | 21 | 841 | LVDGVPSR | Ig kappa chain V-V region T1 | *Mus musculus* |
| Q8C7V3 | 12 | 13 | 21 | 849 | YSQEPVK | U3 small nucleolar RNA-associated protein 15 homolog | *Mus musculus* |
| P48966 | 16 | 19 | 21 | 827 | MEVPPQK | M-phase inducer phosphatase 2 | *Rattus norvegicus* |
| **fraction 31** | | | | | | | |
| gi\|3318722 | 52 | 36 | 183  110  95  71  108  86 | 2210  2282  1044  841  1515  1051 | R.LGEHNIDVLEGNEQFINAAK.I  K.IITHPNFNGNTLDNDIMLIK.L  K.LSSPATLNSR.V  R.VATVSLPR.S  K.SSGSSYPSLLQCLK.A  K.APVLSDSSCK.S | Chain E, Leech-Derived Tryptase Inhibitor | *Sus scrofa* |
| gi\|201006 | 16 | 12 | 114  56 | 1167  842 | R.HVGDLGNVTAGK.D  R.TMVVHEK.Q | Cu/Zn-superoxide dismutase | *Mus musculus* |
| gi\|122649 | 3 | 8 | 119 | 1230 | K.VNVDDVGGEALGR.L | Hemoglobin subunit beta | *Nycticebus coucang* |
| gi\|7949005 | 2 | 8 | 83 | 1061 | K.FEVIDKPQS.- | ATP synthase-coupling factor 6, mitochondrial precursor | *Mus musculus* |
| gi\|16554572 | 4 | 12 | 60 | 880 | R.WYLGGSAK.G | Sodium/potassium-transporting ATPase subunit gamma isoform b | *Mus musculus* |
| gi\|432104182 | 23 | 22 | 48 | 855 | LAISSLPR | F-box/WD repeat-containing protein 10 | *Myotis davidii* |
| **gi\|521028001** | **14** | **11** | **47** | **1152** | **KDIEFYIPK** | **Hyaluronidase PH-20*** | ***Myotis brandtii*** |
| gi\|655846629 | 13 | 15 | 47 | 827 | VGTAEPR | C2 calcium-dependent domain-containing protein 4D-like, partial | *Oryctolagus cuniculus* |
| gi\|37675525 | 15 | 18 | 44 | 798 | VPEVDIK | AHNAK, partial | *Mus musculus* |
| gi\|226437589 | 47 | 16 | 42 | 1803 | TPEEEPLNLEGLVAHR | Tensin 1 isoform a | *Mus musculus* |
| gi\|759101819 | 13 | 13 | 41 | 757 | KSPADLK | Melanoma-associated antigen F1 | *Pteropus vampyrus* |
| gi\|528769852 | 21 | 15 | 41 | 874 | ELQMPNK + Oxidation (M) | Solute carrier organic anion transporter family member 2B1 | *Camelus ferus* |
| gi\|14318722 | 26 | 9 | 38 | 1410 | GAVDAAVPTNIIAAK | ATPase, H+ transporting, lysosomal V1 subunit H | *Mus musculus* |
| gi\|359322085 | 31 | 15 | 36 | 1216 | IPPSIPPGVPSR | Dynamin-2 isoform X11 | *Canis lupus familiaris* |
| gi\|528766190 | 6 | 10 | 34 | 752 | MKCGLR | Proteasome-associated protein ECM29-like protein | *Camelus ferus* |
| gi\|1196614 | 59 | 15 | 33 | 1961 | QVQLQIPGAELVKPGASVK | Immunoglobulin heavy chain, partial | *Mus musculus domesticus* |
| gi\|50054054 | 18 | 8 | 32 | 1249 | KKPLVDQMFK + Oxidation (M) | Follistatin-related protein 5 precursor | *Mus musculus* |
| gi\|830220972 | 18 | 12 | 32 | 1000 | VDTGGVQVAR | GTP-binding protein Rheb | *Condylura cristata* |
| gi\|884944376 | 14 | 12 | 32 | 1277 | LSLAQLENLCK | Exportin-7 isoform X1 | *Cavia porcellus* |
| gi\|987936065 | 7 | 8 | 31 | 776 | LEFPSGK | Protein APCDD1-like | *Myotis davidii* |
| gi\|852790143 | 18 | 18 | 31 | 849 | VPTAPIPR | Collagen alpha-3(IX) chain | *Dipodomys ordii* |
| gi\|585682264 | 11 | 9 | 30 | 1075 | MMLTNQNPK | Centrosomal protein of 162 kDa | *Elephantulus edwardii* |
| gi\|432105468 | 11 | 8 | 30 | 1000 | VPKTAENSR | Peptidyl-prolyl cis-trans isomerase A | *Myotis davidii* |
| gi\|521022217 | 29 | 15 | 30 | 1235 | SKWHIPVPSGK | Transmembrane protein 87A | *Myotis brandtii* |
| gi\|194206109 | 23 | 17 | 30 | 942 | SLIPAEGTR | RNA-binding protein 34 | *Equus caballus* |
| **fraction 34** | | | | | | | |
| gi\|3318722 | 48 | 36 | 177  93  77  108  98 | 2210  1044  841  1515  1051 | R.LGEHNIDVLEGNEQFINAAK.I  K.LSSPATLNSR.V  R.VATVSLPR.S  K.SSGSSYPSLLQCLK.A  K.APVLSDSSCK.S | Chain E, Leech-Derived Tryptase Inhibitor | *Sus scrofa* |
| gi\|505855613 | 27 | 39 | 186  79  97  87 | 2062  1787  1803  1061 | R.QSSGGPVDTGPEYQQELDR.E  K.ADMNTFPNFTFEEPK.F  K.ADMNTFPNFTFEEPK.F + Oxidation (M)  K.FEVIDKPQS.- | ATP synthase-coupling factor 6, mitochondrial | *Sorex araneus* |
| gi\|201006 | 10 | 12 | 120  53 | 1167  842 | R.HVGDLGNVTAGK.D  R.TMVVHEK.Q | Cu/Zn-superoxide dismutase | *Mus musculus* |
| gi\|33087199 | 13 | 25 | 48 | 764 | SSVVFVK | Lipoprotein lipase, partial | *Sus scrofa* |
| gi\|548454268 | 48 | 18 | 48 | 1699 | TKPADEEMLFIYSR | Acyl-CoA-binding protein | *Capra hircus* |
| gi\|115270960 | 13 | 11 | 46 | 989 | TTTWNDPR | BAG family molecular chaperone regulator 3 | *Mus musculus* |
| gi\|432104182 | 27 | 26 | 45 | 855 | LAISSLPR | F-box/WD repeat-containing protein 10 | *Myotis davidii* |
| gi\|594059187 | 16 | 16 | 44 | 1008 | ELQHWLAI | Sodium-dependent noradrenaline transporter isoform X3 | *Bubalus bubalis* |
| gi\|57094432 | 31 | 20 | 42 | 1061 | EATAEDGELK | Transcription initiation factor TFIID subunit 11 | *Canis lupus familiaris* |
| gi\|560905974 | 9 | 10 | 39 | 788 | IVDTLTK | BPI fold-containing family A member 2 | *Camelus ferus* |
| gi\|674052932 | 10 | 10 | 39 | 875 | EDLDSLGK | Leucine-rich repeat-containing G-protein coupled receptor 5 isoform X1 | *Nannospalax galili* |
| gi\|505853959 | 19 | 7 | 39 | 1535 | LSEDSGVSTNVSVNK | Protein Mis18-alpha | *Sorex araneus* |
| gi\|27356782 | 15 | 14 | 39 | 868 | VIPELNGK | Glyceraldehyde-3-phosphate dehydrogenase | *Meriones unguiculatus* |
| gi\|28175136 | 19 | 13 | 38 | 1179 | LLAVIEEQHK | Slc38a10 protein, partial | *Mus musculus* |
| gi\|545557338 | 16 | 20 | 36 | 714 | LASLVGR | Odorant-binding protein-like | *Canis lupus familiaris* |
| gi\|852790143 | 30 | 30 | 36 | 849 | VPTAPIPR | Collagen alpha-3(IX) chain | *Dipodomys ordii* |
| gi\|505775335 | 44 | 15 | 36 | 1262 | NAEKSTGGGGIGSK | DnaJ homolog subfamily C member 2 isoform X1 | *Sorex araneus* |
| gi\|836714455 | 49 | 16 | 35 | 1787 | EVSIEERLGALDIDTK | WD repeat-containing protein 43 | *Sorex araneus* |
| gi\|731505870 | 14 | 16 | 34 | 812 | DEVPEPK | Microtubule-associated protein 1A | *Loxodonta africana* |
| gi\|830220972 | 17 | 11 | 32 | 1000 | VDTGGVQVAR | GTP-binding protein Rheb | *Condylura cristata* |
| gi\|27658051 | 27 | 17 | 30 | 1042 | LEAAGVAEQR | MHC class I antigen | *Equus caballus* |
| **fraction 39** | | | | | | | |
| Q3T140 | 87 | 83 | 50  105  111  39  77  85  92  77  57  54  47  44 | 2220  1636  2439  2455  1903  1460  1143  1015  1031  2448  2464  1451 | R.LQSQKGVQGIIVVNTEGIPIK.S  K.GVQGIIVVNTEGIPIK.S  K.STMDNPTTTQYANLMHNFILK.A  K.STMDNPTTTQYANLMHNFILK.A + Oxidation (M)  R.STVREIDPQNDLTFLR.I  R.EIDPQNDLTFLR.I  K.KNEIMVAPDK.D  K.NEIMVAPDK.D  K.NEIMVAPDK.D + Oxidation (M)  K.NEIMVAPDKDYFLIVIQNPTE.-  K.NEIMVAPDKDYFLIVIQNPTE.- + Oxidation (M)  K.DYFLIVIQNPTE.- | Dynein light chain roadblock-type 1 | *Bos taurus* |
| P18203 | 19 | 25 | 78  105  71 | 1314  1533  1549 | M.GVQVETISPGDGR.T  R.GWEEGVAQMSVGQR.A  R.GWEEGVAQMSVGQR.A + Oxidation (M) | Peptidyl-prolyl cis-trans isomerase FKBP1A | *Bos taurus* |
| Q3ZBZ8 | 18 | 8 | 86  71  80  75 | 1889  1001  1136  1100 | R.AMADPEVQQIMSDPAMR.L  R.LILEQMQK.D  K.DPQALSEHLK.N  K.LMDVGLIAIR.- | Stress-induced-phosphoprotein 1 | *Bos taurus* |
| P54149 | 15 | 10 | 92  86 | 1166  1616 | K.IVSPQEALPGR.K  K.VFWENHDPTQGMR.Q | Mitochondrial peptide methionine sulfoxide reductase | *Bos taurus* |
| Q3YIX4 | 8 | 10 | 123 | 1949 | K.GNDISSGTVLSDYVGSGPPK.G | Phosphatidylethanolamine-binding protein 1 | *Canis lupus familiaris* |
| Q6P7Q4 | 17 | 25 | 78  54  44  57  65 | 1264  1028  900  976  2288 | K.DFLLQQTMLR.I  K.KSLDFYTR.V  K.SLDFYTR.V  K.RFEELGVK.F  K.GLAFVQDPDGYWIEILNPNK.M | Lactoylglutathione lyase | *Rattus norvegicus* |
| Q0VCX2 | 8 | 7 | 39  63  72  68 | 985  1316  1974  1397 | R.LTPEEIER.M  R.NELESYAYSLK.N  K.IEWLESHQDADIEDFK.A  K.ELEEIVQPIISK.L | 78 kDa glucose-regulated protein | *Bos taurus* |
| Q6X9Z5 | 5 | 10 | 76 | 1256 | K.NIEDVIAQGIGK.L | 60S acidic ribosomal protein P2 | *Equus caballus* |
| Q3SZ68 | 5 | 21 | 73 | 1994 | R.GTLSDEHAGVISVLAQQAAK.L | Ragulator complex protein LAMTOR5 | *Bos taurus* |
| Q2NKV2 | 5 | 13 | 76 | 1229 | R.ILDLIDDAWR.E | Anaphase-promoting complex subunit 13 | *Bos taurus* |
| Q3T0E0 | 5 | 11 | 81 | 833 | K.AVSYLGPK.- | Copper transport protein ATOX1 | *Bos taurus* |
| P02049 | 5 | 15 | 102  41 | 1300  1274 | K.VNVDDVGGEALGR.L  R.LLVVYPWTQR.F | Hemoglobin subunit beta | *Nycticebus coucang* |
| P62077 | 3 | 21 | 65  56 | 1207  832 | R.FIDTTLAITGR.F  R.FAQIVQK.G | Mitochondrial import inner membrane translocase subunit Tim8 B | *Mus musculus* |
| B0VYY2 | 2 | 10 | 50  62 | 770  991 | K.IIDAALR.A  R.LNDFASAVR.I | Cytochrome c oxidase subunit 5A, mitochondrial | *Nycticebus coucang* |
| P11751 | 2 | 10 | 57 | 1492 | K.VGGQAGDYGAEALER.M | Hemoglobin subunit alpha | *Megaderma lyra* |
| Q8WN94 | 1 | 9 | 67 | 1060 | K.WDAWNELK.G | Acyl-CoA-binding protein | *Oryctolagus cuniculus* |
| P21571 | 2 | 8 | 41 | 1061 | K.FEVLDKPQS.- | ATP synthase-coupling factor 6, mitochondrial | *Rattus norvegicus* |
| **P00592** | **1** | **4** | **31** | **819** | **R.ALWQFR.S** | **Phospholipase A2, major isoenzyme** | ***Sus scrofa*** |
| P35433 | 13 | 12 | 29 | 931 | ELEESGIR | Amidophosphoribosyltransferase | *Rattus norvegicus* |
| Q62924 | 20 | 22 | 28 | 822 | ELLFSSK | A-kinase anchor protein 11 | *Rattus norvegicus* |
| **Q9Z0F8** | **12** | **9** | **27** | **1095** | **SEDIKDFSR** | **Disintegrin and metalloproteinase domain-containing protein 17** | ***Mus musculus*** |
| Q9QYI6 | 28 | 10 | 27 | 1796 | EIAEAYETLSDANSRK | DnaJ homolog subfamily B member 9 | *Mus musculus* |
| Q920B9 | 7 | 14 | 27 | 705 | MIDAIK + Oxidation (M) | FACT complex subunit SPT16 | *Mus musculus* |
| Q148N0 | 12 | 14 | 27 | 969 | SWDIFFR | 2-oxoglutarate dehydrogenase, mitochondrial | *Bos taurus* |
| Q6P5D4 | 22 | 12 | 26 | 1215 | IANLQESLLSK | Centrosomal protein of 135 kDa | *Mus musculus* |
| Q9JL60 | 15 | 17 | 26 | 918 | EMEELLR | Glucocorticoid modulatory element-binding protein 1 | *Mus musculus* |
| Q95MM9 | 5 | 8 | 26 | 772 | ILESRR | Signaling lymphocytic activation molecule | *Canis lupus familiaris* |
| P52552 | 13 | 10 | 26 | 1069 | LVQGFQYTD | Peroxiredoxin-2 (Fragment) | *Sus scrofa* |
| P46892 | 11 | 10 | 25 | 845 | DIGTPSEK | Cyclin-dependent kinase 11B | *Rattus norvegicus* |
| Q9CXF4 | 13 | 12 | 25 | 825 | GGLSHSLR | TBC1 domain family member 15 | *Mus musculus* |
| Q9EPQ8 | 20 | 14 | 25 | 1019 | EAMTGRVEK | Transcription factor 20 | *Mus musculus* |
| Q91WR3 | 13 | 16 | 25 | 863 | RMAFLAR | Activating signal cointegrator 1 complex subunit 2 | *Mus musculus* |
| Q9ERA5 | 15 | 10 | 24 | 1051 | KEYDAVAEK | Structural maintenance of chromosomes protein 4 (Fragment) | *Microtus arvalis* |
| Q148E1 | 12 | 16 | 24 | 761 | GCQLAPK | Apoptogenic protein 1, mitochondrial | *Bos taurus* |
| Q0VCX2 | 13 | 12 | 24 | 985 | LTPEEIER | 78 kDa glucose-regulated protein | *Bos taurus* |
| P54279 | 9 | 10 | 24 | 931 | HELSYRK | Mismatch repair endonuclease PMS2 | *Mus musculus* |
| Q9DB41 | 25 | 13 | 24 | 1165 | MSSQDLSISAK | Mitochondrial glutamate carrier 2 | *Mus musculus* |
| Q9JLF7 | 11 | 11 | 24 | 949 | MLNLAFNK | Toll-like receptor 5 | *Mus musculus* |
| P12263 | 19 | 7 | 23 | 1427 | ISALGKSAAGPLASGK | Coagulation factor VIII | *Sus scrofa* |
| Q6DFX2 | 24 | 11 | 23 | 1355 | LANEQIQNAGGLK | Anthrax toxin receptor 2 | *Mus musculus* |
| O08550 | 26 | 13 | 23 | 1342 | TSSPLRTSPQLR | Histone-lysine N-methyltransferase 2B | *Mus musculus* |
| Q99N92 | 14 | 14 | 22 | 785 | AAAALTLR | 39S ribosomal protein L27, mitochondrial | *Mus musculus* |
| Q8BJS8 | 32 | 16 | 22 | 1328 | AMIDIILLPSDK | Mdm2-binding protein | *Mus musculus* |
| Q9MZ03 | 12 | 13 | 22 | 895 | NMHPELR | ADP-ribosyl cyclase/cyclic ADP-ribose hydrolase 1 | *Oryctolagus cuniculus* |
| Q28730 | 15 | 13 | 22 | 897 | RNGPEGLR | Intercellular adhesion molecule 5 | *Oryctolagus cuniculus* |
| Q8CG48 | 12 | 14 | 22 | 865 | YEALENK | Structural maintenance of chromosomes protein 2 | *Mus musculus* |
| Q3UMY5 | 18 | 12 | 21 | 933 | KETLSSAAK | Echinoderm microtubule-associated protein-like 4 | *Mus musculus* |
| Q5E9I1 | 14 | 8 | 21 | 1317 | ITHLPTIPETVP | Cyclin-G1 | *Bos taurus* |
| Q8BRB7 | 32 | 11 | 21 | 1652 | RPVAGERGQLLELSK | Histone acetyltransferase KAT6B | *Mus musculus* |
| Q2KIE4 | 14 | 9 | 21 | 937 | MSAEDIEK + Oxidation (M) | Malignant T-cell-amplified sequence 1 | *Bos taurus* |
| P34943 | 8 | 10 | 21 | 855 | AIEVLRR | NADH dehydrogenase [ubiquinone] 1 alpha subcomplex subunit 9, mitochondrial | *Bos taurus* |
| A5PJU9 | 12 | 11 | 21 | 939 | MQEMLEK + 2 Oxidation (M) | Septin-1 | *Bos taurus* |
| **fraction 40** | | | | | | | |
| O89106 | 39 | 36 | 51  84  94  81  70 | 1612  1149  2365  2381  1093 | R.FGQHLIKPSVVFLK.T  K.TELSFALVNR.K  K.HFQGTSITFSMQDGPEAGQTVK.H  K.HFQGTSITFSMQDGPEAGQTVK.H + Oxidation (M)  K.HVHVHVLPR.K | Bis(5'-adenosyl)-triphosphatase | *Mus musculus* |
| Q6P7Q4 | 44 | 33 | 75  77  43  56  104  73  43  63  72 | 1264  1280  1028  900  1395  1962  976  820  2288 | K.DFLLQQTMLR.I  K.DFLLQQTMLR.I + Oxidation (M)  K.KSLDFYTR.V  K.SLDFYTR.V  K.FSLYFLAYEDK.N  K.FSLYFLAYEDKNDIPK.D  K.RFEELGVK.F  R.FEELGVK.F  K.GLAFVQDPDGYWIEILNPNK.M | Lactoylglutathione lyase | *Rattus norvegicus* |
| Q3YIX4 | 20 | 21 | 91  30  153  53 | 1439  1455  1949  1091 | R.EWHHFLVVNMK.G  R.EWHHFLVVNMK.G + Oxidation (M)  K.GNDISSGTVLSDYVGSGPPK.G  K.CDEPILSNR.S | Phosphatidylethanolamine-binding protein 1 | *Canis lupus familiaris* |
| Q3T140 | 15 | 72 | 93  66  79  35 | 1636  2439  1460  2448 | K.GVQGIIVVNTEGIPIK.S  K.STMDNPTTTQYANLMHNFILK.A  R.EIDPQNDLTFLR.I  K.NEIMVAPDKDYFLIVIQNPTE.- | Dynein light chain roadblock-type 1 | *Bos taurus* |
| P18203 | 13 | 40 | 75  42  106 | 1314  1939  1533 | M.GVQVETISPGDGR.T  K.RGQTCVVHYTGMLEDGK.K  R.GWEEGVAQMSVGQR.A | Peptidyl-prolyl cis-trans isomerase FKBP1A | *Bos taurus* |
| B0VYY2 | 14 | 20 | 92  74  86 | 1632  770  991 | K.GMNTLVGYDLVPEPK.I  K.IIDAALR.A  R.LNDFASAVR.I | Cytochrome c oxidase subunit 5A, mitochondrial | *Nycticebus coucang* |
| Q9CWM4 | 19 | 54 | 45  115  37  52  84  32 | 906  1312  2731  865  1222  765 | K.AFTELQAK.V  K.LADIQIEQLNR.T  K.HAHLTDTEIMTLVDETNMYEGVGR.M  R.MFILQSK.E  K.EVIHNQLLEK.Q  R.EMLMAR.R + Oxidation (M) | Prefoldin subunit 1 | *Mus musculus* |
| P54149 | 13 | 10 | 92  62 | 1166  1616 | K.IVSPQEALPGR.K  K.VFWENHDPTQGMR.Q | Mitochondrial peptide methionine sulfoxide reductase | *Bos taurus* |
| Q9JLV1 | 18 | 6 | 54  65  29  98  55 | 800  1302  1318  1413  829 | K.VEAILEK.V  K.YLMIEEYLTK.E K.YLMIEEYLTK.E + Oxidation (M)  K.ELLALDSVDPEGR.A  K.VQTILEK.L | BAG family molecular chaperone regulator | *Mus musculus* |
| H6BDU4 | 9 | 14 | 94  31 | 1167  989 | R.HVGDLGNVTAGK.D  R.LACGVIGIAQ.- | Superoxide dismutase [Cu-Zn] | *Camelus dromedarius* |
| Q9CXP8 | 5 | 19 | 54  74 | 728  772 | R.LVEQLK.L  K.LEAGVER.I | Guanine nucleotide-binding protein G(I)/G(S)/G(O) subunit gamma-10 | *Mus musculus* |
| P02102 | 9 | 14 | 60  29 | 1274  1166 | R.LLVVYPWTQR.F  K.LVSGVATALAHK.Y | Hemoglobin subunit epsilon-1 | *Capra hircus* |
| Q3T0E0 | 1 | 11 | 77 | 833 | K.AVSYLGPK.- | Copper transport protein ATOX1 | *Bos taurus* |
| P62959 | 4 | 11 | 57 | 1388 | K.AQVAQPGGDTIFGK.I | Histidine triad nucleotide-binding protein 1 | *Rattus norvegicus* |
| Q8WN94 | 5 | 9 | 51 | 1060 | K.WDAWNELK.G | Acyl-CoA-binding protein | *Oryctolagus cuniculus* |
| P11751 | 2 | 10 | 42 | 1492 | K.VGGQAGDYGAEALER.M | Hemoglobin subunit alpha | *Megaderma lyra* |
| Q8R151 | 18 | 19 | 31 | 720 | MAEKVK + Oxidation (M) | NFX1-type zinc finger-containing protein 1 | *Mus musculus* |
| Q3V3V9 | 12 | 15 | 30 | 786 | DRLVER | Capping protein, Arp2/3 and myosin-I linker protein 2 | *Mus musculus* |
| P97679 | 12 | 12 | 30 | 829 | LVESAALK | DNA mismatch repair protein Mlh1 | *Rattus norvegicus* |
| Q8HYY4 | 15 | 11 | 30 | 861 | LMRAAER + Oxidation (M) | Uveal autoantigen with coiled-coil domains and ankyrin repeats protein | *Bos taurus* |
| P43023 | 2 | 11 | 29 | 1120 | K.VLSRSMASAAK.G | Cytochrome c oxidase subunit 6A2, mitochondrial | *Mus musculus* |
| Q5EAD4 | 17 | 18 | 29 | 861 | MERATVR | Short/branched chain specific acyl-CoA dehydrogenase, mitochondrial | *Bos taurus* |
| P54279 | 7 | 8 | 29 | 931 | HELSYRK | Mismatch repair endonuclease PMS2 | *Mus musculus* |
| P00586 | 20 | 13 | 28 | 1048 | TVSVLNGGFR | Thiosulfate sulfurtransferase | *Bos taurus* |
| A6QM06 | 10 | 14 | 28 | 932 | MELADLNK | Sterol regulatory element-binding protein cleavage-activating protein | *Bos taurus* |
| **P14422** | **6** | **6** | **27** | **1002** | **FAKFLSYK** | **Phospholipase A2** | ***Oryctolagus cuniculus*** |
| O08550 | 19 | 9 | 27 | 1342 | TSSPLRTSPQLR | Histone-lysine N-methyltransferase 2B | *Mus musculus* |
| Q9D361 | 12 | 11 | 27 | 870 | IPSVTLNK | U11/U12 small nuclear ribonucleoprotein 48 kDa protein | *Mus musculus* |
| Q0VCR8 | 25 | 24 | 27 | 829 | LAALSSLR | Exocyst complex component 3-like protein | *Bos taurus* |
| Q9D799 | 17 | 15 | 27 | 887 | ELEAVLSK | Methionyl-tRNA formyltransferase, mitochondrial | *Mus musculus* |
| Q9DB41 | 15 | 8 | 26 | 1165 | MSSQDLSISAK | Mitochondrial glutamate carrier 2 | *Mus musculus* |
| Q9JLF7 | 10 | 10 | 26 | 949 | MLNLAFNK | Toll-like receptor 5 | *Mus musculus* |
| P02680 | 15 | 13 | 26 | 988 | KMVEEILK | Fibrinogen gamma chain | *Rattus norvegicus* |
| Q8BJS8 | 26 | 13 | 26 | 1328 | AMIDIILLPSDK | Mdm2-binding protein | *Mus musculus* |
| P35479 | 18 | 16 | 26 | 969 | VKPQLEEK | Leukocyte cysteine proteinase inhibitor 1 | *Sus scrofa* |
| A4K436 | 11 | 14 | 26 | 702 | KESDPK | Regulator of telomere elongation helicase 1 | *Bos taurus* |
| Q9WTZ8 | 17 | 12 | 26 | 1002 | ESKVEQGVK | Protein BEX2 | *Mus musculus* |
| Q924Y8 | 20 | 21 | 25 | 789 | QAVSEEK | Probable G-protein coupled receptor 149 | *Rattus norvegicus* |
| Q8VI38 | 9 | 10 | 25 | 906 | RMETINK + Oxidation (M) | Globoside alpha-1,3-N-acetylgalactosaminyltransferase 1 | *Mus musculus* |
| Q8BQ48 | 20 | 10 | 25 | 1296 | SDATVSSDNMDR | Centrosomal protein of 295 kDa | *Mus musculus* |
| P46892 | 14 | 12 | 25 | 845 | DIGTPSEK | Cyclin-dependent kinase 11B | *Rattus norvegicus* |
| P24643 | 13 | 17 | 24 | 951 | WEVDEMK + Oxidation (M) | Calnexin | *Canis lupus familiaris* |
| Q27975 | 24 | 18 | 24 | 946 | SAVEDEGLK | Heat shock 70 kDa protein 1A | *Bos taurus* |
| Q9XTA2 | 12 | 18 | 24 | 905 | AFVEAQNK | Prolyl endopeptidase | *Bos taurus* |
| Q9JJA2 | 15 | 17 | 24 | 881 | LLDRLPR | Conserved oligomeric Golgi complex subunit 8 | *Mus musculus* |
| Q99P69 | 15 | 9 | 23 | 952 | MKSDISEK + Oxidation (M) | Kinetochore protein Nuf2 | *Mus musculus* |
| Q3MHM6 | 15 | 14 | 23 | 829 | ALLSAVTR | Catenin alpha-1 | *Bos taurus* |
| Q8SPJ1 | 9 | 13 | 23 | 813 | MVPLLNK | Junction plakoglobin | *Bos taurus* |
| P11708 | 16 | 12 | 23 | 992 | EVGVYEAVK | Malate dehydrogenase, cytoplasmic | *Sus scrofa* |
| Q8K0Z7 | 20 | 12 | 23 | 992 | NGGMMAEGAR | Translational activator of cytochrome c oxidase 1 | *Mus musculus* |
| O02810 | 22 | 17 | 22 | 1102 | LAPEREFIK | Phosphatidylinositol 4-kinase beta | *Bos taurus* |
| D3ZZL9 | 15 | 14 | 22 | 737 | EMESVK + Oxidation (M) | GRIP and coiled-coil domain-containing protein 2 | *Rattus norvegicus* |
| P61603 | 21 | 16 | 21 | 923 | MAGQAFRK + Oxidation (M) | 10 kDa heat shock protein, mitochondrial | *Bos taurus* |
| A1XQU3 | 16 | 17 | 21 | 717 | KITTAGK | 60S ribosomal protein L14 | *Sus scrofa* |
| P83095 | 15 | 17 | 21 | 949 | HYEKDMK | Serine beta-lactamase-like protein LACTB, mitochondrial | *Bos taurus* |

*hyaluronidase does not display toxic activity but is a toxin spreading factor commonly found in animal venoms
