# Supplementary material for: A new, widespread venomous mammal species: hemolytic activity of Sorex araneus venom is similar to that of Neomys fodiens venom
Source: Zoological Lett. 2022 Jun 7;8:7. doi: 10.1186/s40851-022-00191-5 (PMC9172195; doi:10.1186/s40851-022-00191-5)
Supplement: Supplementary file 2 — Additional file 2: Table A2. Protein identification in the extract from venom glands of the common shrew, Sorex araneus, based on tandem mass spectrometry analysis. Toxins are shown in bold. [file 40851_2022_191_MOESM2_ESM.docx]

**A new, widespread venomous mammal species: hemolytic activity of *Sorex araneus* venom is similar to that of *Neomys fodiens* venom**

Krzysztof Kowalski^1*^, Paweł Marciniak^2^ and Leszek Rychlik^3^

^1^Department of Vertebrate Zoology and Ecology, Institute of Biology, Faculty of Biological and Veterinary Sciences, Nicolaus Copernicus University, Lwowska 1, Toruń, 87-100, Poland, e-mail: k.kowalski@umk.pl,: tel. +48 56 611 4910

^2^Department of Animal Physiology and Developmental Biology, Institute of Experimental Biology, Faculty of Biology, Adam Mickiewicz University, Uniwersytetu Poznańskiego 6, Poznań, 61-614, Poland, e-mail: pmarcin@amu.edu.pl, tel.: +48 61 829 5926

^3^Department of Systematic Zoology, Institute of Environmental Biology, Faculty of Biology, Adam Mickiewicz University, Uniwersytetu Poznańskiego 6, Poznań, 61-614, Poland, e-mail: leszek.rychlik@amu.edu.pl, tel.: +48 61 829 5751

*Correspondence: k.kowalski@umk.pl (K.K.)

**Table A2** Protein identification in the extract from venom glands of the common shrew *Sorex araneus* based on tandem mass spectrometry analysis. Toxins are shown in bold.

| **Accession** | **Matched peptides** | **Protein sequence coverage [%]** | **Ion score** | **m/z** | **Identified peptides** | **Protein name** | **Organism** |
| --- | --- | --- | --- | --- | --- | --- | --- |
| **whole extract** | | | | | | | |
| P63312 | 7 | 34 | 56  37  48  77 | 862  734  875  1031 | K.KTETQEK.N  K.TETQEK.N  K.ETIEQEK.R  K.ETIEQEKR.S | Thymosin beta-10 | *Rattus norvegicus* |
| P07107 | 76 | 39 | 53  123  73 | 1987  1991  2007 | K.TKPADEEMLFIYSHYK.Q + Oxidation (M)  K.QATVGDINTERPGMLDFK.G  K.QATVGDINTERPGMLDFK.G + Oxidation (M) | Acyl-CoA-binding protein | *Bos taurus* |
| P18203 | 23 | 25 | 75  113  118 | 1314  1533  1549 | M.GVQVETISPGDGR.T  R.GWEEGVAQMSVGQR.A  R.GWEEGVAQMSVGQR.A + Oxidation (M) | Peptidyl-prolyl cis-trans isomerase FKBP1A | *Bos taurus* |
| Q2KJ32 | 33 | 19 | 104  82  78  78  43  84  60  70 | 2413  1837  1104  1411  1050  1263  2546  1302 | K.GTWEQPGGAAPMGYDFWYQPR.H  R.HNVMISTEWAAPNVLR.D  K.NQGGTWSVEK.V  K.GGPVQVLEDQELK.C  K.QFYPDLIR.E  K.LNPNFLVDFGK.E  K.LNPNFLVDFGKEPLGPALAHELR.Y  K.EPLGPALAHELR.Y | Selenium-binding protein 1 | *Bos taurus* |
| Q2KJG2 | 27 | 68 | 53  100  66  80 | 901  1587  840  2752 | K.ITLTSDPR.L  K.VLSVPESTPFTAVLK.F  K.FAAEEFK.V  K.VPAATSAIITNDGIGINPAQTAGNVFLK.H | Ubiquitin-fold modifier 1 | *Bos taurus* |
| P04906 | 20 | 13 | 93  82  65 | 1351  1276  736 | M.PPYTIVYFPVR.G  R.MLLADQGQSWK.E  R.SLGLYGK.D | Glutathione S-transferase P | *Rattus norvegicus* |
| Q9DBJ1 | 22 | 40 | 76  61  102  61  34  36  54  118  56 | 1311  1058  1683  1150  3038  2114  2130  1102  1118 | R.HGESAWNLENR.F  R.HYGGLTGLNK.A  R.ALPFWNEEIVPQIK.E  R.VLIAAHGNSLR.G  K.HLEGLSEEAIMELNLPTGIPIVYELDK.N + Oxidation (M)  K.NLKPIKPMQFLGDEETVR.K  K.NLKPIKPMQFLGDEETVR.K + Oxidation (M)  R.KAMEAVAAQGK.V  R.KAMEAVAAQGK.V + Oxidation (M) | Phosphoglycerate mutase 1 | *Mus musculus* |
| B3EWE1 | 27 | 43 | 67  88  75  64  60 | 998  1793  844  2186  817 | R.TFASFPTTK.T  K.TYFPHFDLSPGSAQVK.G  K.KVADALTK.A  K.AVGSLDDLPGALSALSDLHAHK.L  R.VDPVNFK.L | Hemoglobin subunit alpha | *Blarina brevicauda* |
| P21571 | 25 | 21 | 62  54  91 | 827  882  1061 | K.ELDPVQK.L  K.LKQMYGK.G + Oxidation (M)  K.FEVLDKPQS.- | ATP synthase-coupling factor 6, mitochondrial | *Rattus norvegicus* |
| Q3MHL6 | 18 | 18 | 60  35  70  92 | 975  991  844  1429 | K.SHLMYAVR.E  K.SHLMYAVR.E + Oxidation (M)  R.EEVEVLK.E  K.NSQLEQENNLLK.T | TSC22 domain family protein 1 | *Bos taurus* |
| Q3SZR3 | 36 | 11 | 52  64 | 1145  1686 | K.WFYIGSAFR.N  K.DACGPLEKQHEEER.K | Alpha-1-acid glycoprotein | *Bos taurus* |
| Q3T140 | 24 | 16 | 53 | 1636 | K.GVQGIIVVNTEGIPIK.S | Dynein light chain roadblock-type 1 | *Bos taurus* |
| Q2KIV2 | 12 | 34 | 52  130  77 | 970  1789  810 | K.EFLGTYNK.L  R.FQEYHIQQNEALAAK.A  K.AGLLGQPR.- | Mitochondrial import inner membrane translocase subunit Tim9 | *Bos taurus* |
| Q2EN75 | 16 | 15 | 62  58  58 | 730  876  892 | K.ELTIGAK.L  K.LMDDLDR.N  K.LMDDLDR.N + Oxidation (M) | Protein S100-A6 | *Sus scrofa* |
| Q9D0S9 | 6 | 12 | 125 | 2219 | R.ISQAEEDDQQLLGHLLLVAK.K | Histidine triad nucleotide-binding protein 2, mitochondrial | *Mus musculus* |
| Q3ZC22 | 7 | 14 | 86 | 1295 | K.FQTMSDQIIGR.I | Heat shock factor-binding protein 1 | *Bos taurus* |
| Q8WNN6 | 10 | 18 | 113  53  58 | 1167  842  989 | R.HVGDLGNVTAGK.D  R.TMVVHEK.R  R.LACGVIGIAQ.- | Superoxide dismutase [Cu-Zn] | *Canis lupus familiaris* |
| P58044 | 13 | 14 | 44  61  34  46 | 769  1353  790  1067 | K.LLLQQR.S  K.NVTLNPDPNEIK.S  K.LTPWFK.I  K.IIADTFLFK.W | Isopentenyl-diphosphate Delta-isomerase 1 | *Mus musculus* |
| Q3ZBS8 | 7 | 22 | 65  74 | 1353  1194 | R.FIDTSQFILNR.L  K.SKPVFSESLSD.- | Mitochondrial import inner membrane translocase subunit Tim8 A | *Bos taurus* |
| Q5S3G4 | 4 | 8 | 82 | 1200 | K.GLDPYNILAPK.A | Cytochrome c oxidase subunit 5B, mitochondrial | *Sus scrofa* |
| Q1ZZU7 | 4 | 9 | 76  66 | 1273  1289 | M.PMFVVNTNVPR.A  M.PMFVVNTNVPR.A + Oxidation (M) | Macrophage migration inhibitory factor | *Ovis aries* |
| Q2NKU6 | 3 | 16 | 74 | 1886 | K.ERPPNPIEFLASYLLK.N | Protein dpy-30 homolog | *Bos taurus* |
| Q6P7Q4 | 3 | 20 | 51  42  74 | 1264  976  2288 | K.DFLLQQTMLR.I  K.RFEELGVK.F  K.GLAFVQDPDGYWIEILNPNK.M | Lactoylglutathione lyase | *Rattus norvegicus* |
| Q56K04 | 2 | 11 | 54 | 932 | R.GGAESHTFK.- | Cysteine-rich protein 1 | *Bos taurus* |
| Q3SZ18 | 2 | 10 | 33  67 | 1178  1277 | K.SIPMTVDFIR.L  K.VIGGDDLSTLTGK.N | Hypoxanthine-guanine phosphoribosyltransferase | *Bos taurus* |
| Q9D6Y7 | 2 | 9 | 48  36 | 867  1632 | K.TGHAEVVR.V  K.VFWENHDPTQGMR.Q + Oxidation (M) | Mitochondrial peptide methionine sulfoxide reductase | *Mus musculus* |
| Q9D8Z2 | 1 | 17 | 32 | 1356 | K.GDGSGDPCTDLFK.R | TP53-regulated inhibitor of apoptosis 1 | *Mus musculus* |
| P26260 | 18 | 13 | 30 | 1086 | EKEATTRPR | Syndecan-1 | *Rattus norvegicus* |
| Q08DK5 | 18 | 33 | 30 | 760 | LDLDASK | Endophilin-B2 | *Bos taurus* |
| Q865B6 | 24 | 20 | 29 | 800 | QVSPGSAR | Peroxisome proliferator-activated receptor gamma coactivator 1-alpha | *Sus scrofa* |
| Q3SZE2 | 5 | 10 | 29 | 865 | MFILQSK | Prefoldin subunit 1 | *Bos taurus* |
| Q8VC56 | 17 | 10 | 28 | 1106 | MVDNLSSDVK | E3 ubiquitin-protein ligase RNF8 | *Mus musculus* |
| Q0P565 | 13 | 35 | 28 | 780 | TGWVYR | HD domain-containing protein 2 | *Bos taurus* |
| Q9ERA5 | 10 | 10 | 28 | 748 | LDMINK + Oxidation (M) | Structural maintenance of chromosomes protein 4 (Fragment) | *Microtus arvalis* |
| Q11126 | 11 | 9 | 27 | 1138 | MYPPGCAKVK | Galactoside 3(4)-L-fucosyltransferase | *Bos taurus* |
| Q5H8C4 | 15 | 25 | 27 | 847 | KDMVDIK | Vacuolar protein sorting-associated protein 13A | *Mus musculus* |
| P19803 | 28 | 9 | 27 | 1917 | SIQEIQELDKDDESLR | Rho GDP-dissociation inhibitor 1 | *Bos taurus* |
| O97594 | 21 | 11 | 27 | 1035 | ELEKMTNR + Oxidation (M) | Structural maintenance of chromosomes protein 3 | *Bos taurus* |
| Q5EAD3 | 15 | 16 | 27 | 773 | ARGEADR | Transcription factor NF-E2 45 kDa subunit | *Bos taurus* |
| P15690 | 11 | 14 | 26 | 883 | MLRIPVR | NADH-ubiquinone oxidoreductase 75 kDa subunit, mitochondrial | *Bos taurus* |
| Q91YS8 | 30 | 21 | 26 | 1095 | PGAVEGPRWK | Calcium/calmodulin-dependent protein kinase type 1 | *Mus musculus* |
| Q8CE90 | 23 | 10 | 26 | 1735 | HYEILEVDVASWFK | Dual specificity mitogen-activated protein kinase 7 | *Mus musculus* |
| P97313 | 10 | 11 | 25 | 733 | VMLSLR + Oxidation (M) | DNA-dependent protein kinase catalytic subunit | *Mus musculus* |
| P33705 | 21 | 8 | 25 | 1240 | LSMKEAHAPLK + Oxidation (M) | T-cell surface glycoprotein CD4 | *Canis lupus familiaris* |
| O47558 | 15 | 30 | 25 | 809 | DVLGFLM + Oxidation (M) | Cytochrome b (Fragment) | *Lepus alleni* |
| Q9D483 | 24 | 10 | 25 | 1429 | GPPPPAPTLVINEK | DNA-directed RNA polymerase III subunit RPC3 | *Mus musculus* |
| P62157 | 23 | 10 | 25 | 1265 | DGNGYISAAELR | Calmodulin | *Bos taurus* |
| Q9BEG2 | 12 | 13 | 24 | 821 | AKIDVCK | Interleukin-12 receptor subunit beta-2 | *Bos taurus* |
| Q8BLK9 | 15 | 14 | 24 | 847 | EASAMDPK | Ribosomal protein S6 kinase delta-1 | *Mus musculus* |
| G3MWR8 | 18 | 12 | 24 | 931 | EKDLDGAGK | Protein-methionine sulfoxide oxidase MICAL3 | *Bos taurus* |
| P05126 | 11 | 8 | 24 | 1142 | IYIQAHIER | Protein kinase C beta type | *Bos taurus* |
| Q3MHE4 | 14 | 10 | 24 | 974 | TLESVVLSK | DNA mismatch repair protein Msh2 | *Bos taurus* |
| P04444 | 31 | 16 | 24 | 1114 | KVLTSLGLGVK | Hemoglobin subunit beta-H1 | *Mus musculus* |
| O35314 | 9 | 11 | 24 | 792 | APHLDLK | Secretogranin-1 | *Rattus norvegicus* |
| E9Q6J5 | 35 | 12 | 24 | 1651 | GVGPLMAVGTRGEHDR | Biorientation of chromosomes in cell division protein 1-like 1 | *Mus musculus* |
| P26954 | 14 | 11 | 23 | 911 | EKIPNPSK | Interleukin-3 receptor class 2 subunit beta | *Mus musculus* |
| A3KFM7 | 25 | 16 | 23 | 1015 | AEILGEAADK | Chromodomain-helicase-DNA-binding protein 6 | *Mus musculus* |
| Q330H0 | 30 | 14 | 23 | 1368 | QYSPRSMEAATK | NADH-ubiquinone oxidoreductase chain 2 | *Rhynchonycteris naso* |
| Q9JLM2 | 29 | 9 | 23 | 1960 | QSQSIAKESLTIHEYVK | Natural killer cell receptor 2B4 | *Rattus norvegicus* |
| Q3SZK4 | 16 | 19 | 23 | 939 | MVVAMMMK | Protein TBRG4 | *Bos taurus* |
| P57784 | 26 | 10 | 23 | 1228 | KGGPSAGDVEAIK | U2 small nuclear ribonucleoprotein A' | *Mus musculus* |
| Q8K202 | 14 | 38 | 23 | 703 | MIEIAK | DNA-directed RNA polymerase I subunit RPA49 | *Mus musculus* |
| **Q10741** | **9** | **11** | **23** | **809** | **LYSDGKK** | **Disintegrin and metalloproteinase domain-containing protein 10** | ***Bos taurus*** |
| P23726 | 8 | 7 | 23 | 1020 | YQQDQIVK | Phosphatidylinositol 3-kinase regulatory subunit beta | *Bos taurus* |
| Q6NZP1 | 29 | 17 | 23 | 1175 | LQATEDDKEK | DNA annealing helicase and endonuclease ZRANB3 | *Mus musculus* |
| Q3SX42 | 15 | 12 | 23 | 916 | KTVDDVIK | Charged multivesicular body protein 2b | *Bos taurus* |
| Q9N0J6 | 31 | 22 | 22 | 1030 | EAIITAKER | Fructose-1,6-bisphosphatase isozyme 2 | *Oryctolagus cuniculu* |
| Q8BW10 | 20 | 12 | 22 | 1185 | ELQELLIDGR | RNA-binding protein NOB1 | *Mus musculus* |
| Q8VD75 | 24 | 15 | 22 | 1230 | WLTQIAELEK | Huntingtin-interacting protein 1 | *Mus musculus* |
| Q3SYS1 | 11 | 7 | 22 | 1020 | LAIYGMLPK + Oxidation (M) | 39S ribosomal protein L13, mitochondrial | *Bos taurus* |
| Q60560 | 19 | 16 | 22 | 884 | DRALDPAK | DNA-binding protein SMUBP-2 | *Mesocricetus auratus* |
| P50310 | 16 | 23 | 22 | 831 | DASGNKIK | Phosphoglycerate kinase 1 | *Cricetulus griseus* |
| Q58DC5 | 17 | 7 | 22 | 1122 | QTATILSMDK + Oxidation (M) | GTP-binding protein 1 | *Bos taurus* |
| A5PK65 | 18 | 11 | 22 | 911 | KGTVMTFL + Oxidation (M) | D-dopachrome decarboxylase | *Bos taurus* |
| Q8CFD4 | 17 | 22 | 22 | 960 | EENDVVEK | Sorting nexin-8 | *Mus musculus* |
| P28800 | 29 | 12 | 22 | 1366 | LDNQEPGGQIAPK | Alpha-2-antiplasmin | *Bos taurus* |
| Q91Y47 | 10 | 11 | 21 | 846 | MICAGYK | Coagulation factor XI | *Mus musculus* |
| Q63880 | 14 | 18 | 21 | 902 | EGKDLITK | Carboxylesterase 3A | *Mus musculus* |
| Q9Z2E1 | 22 | 10 | 21 | 1497 | QPVTKFTNHPSNK | Methyl-CpG-binding domain protein 2 | *Mus musculus* |
| O55036 | 23 | 14 | 21 | 1086 | ATESRIPVSK | Telomeric repeat-binding factor 1 (Fragment) | *Cricetulus griseus* |
| P19973 | 15 | 14 | 21 | 841 | AEAAIDPR | Lymphocyte-specific protein 1 | *Mus musculus* |
| Q80WC3 | 16 | 10 | 21 | 1222 | SCIIDKEELK | Trinucleotide repeat-containing gene 18 protein | *Mus musculus* |
| **fraction 23** | | | | | | | |
| P34032 | 69 | 88 | 46  47  34  38  83  89  43  72 | 1245  1652  862  734  1371  1512  875  1348 | M.ADKPDMAEIEK.F  M.ADKPDMAEIEKFDK.S + Oxidation (M)  K.KTETQEK.N  K.TETQEK.N  K.TETQEKNPLPSK.E  K.NPLPSKETIEQEK.Q  K.ETIEQEK.Q  K.ETIEQEKQAGES.- | Thymosin beta-4 | *Oryctolagus cuniculus* |
| B3EWE1 | 5 | 6 | 67 | 998 | R.TFASFPTTK.T | Hemoglobin subunit alpha | *Blarina brevicauda* |
| Q3T0F4 | 2 | 8 | 48 | 1441 | K.AEAGAGSATEFQFR.G | 40S ribosomal protein S10 | *Bos taurus* |
| P83127 | 2 | 58 | 38 | 813 | -.LENGLLR.K | Alpha-N-acetylgalactosaminidase (Fragment) | *Bos indicus* |
| Q60875 | 11 | 7 | 29 | 920 | MTRSAVLK + Oxidation (M) | Rho guanine nucleotide exchange factor 2 | *Mus musculus* |
| Q8BHL3 | 20 | 20 | 28 | 916 | EQQKQEK | TBC1 domain family member 10B | *Mus musculus* |
| P14841 | 14 | 8 | 27 | 1207 | GTHTLTKSSCK | Cystatin-C | *Rattus norvegicus* |
| Q5NUA6 | 8 | 11 | 27 | 772 | QEQLQK | Nuclear factor erythroid 2-related factor 2 | *Bos taurus* |
| P21752 | 9 | 9 | 27 | 862 | KTETQEK | Thymosin beta-10 | *Bos taurus* |
| O46606 | 7 | 12 | 24 | 876 | NPNFEEK | Phospholipase DDHD1 | *Bos taurus* |
| Q9XS63 | 19 | 11 | 23 | 1113 | GLGAERGQQAK | Chromogranin-A | *Equus caballus* |
| O88807 | 17 | 12 | 22 | 1113 | FSDNEDFLK | Protein-arginine deiminase type-4 | *Rattus norvegicus* |
| Q6AYU1 | 17 | 16 | 22 | 949 | YVDANLQK | Mortality factor 4-like protein 1 | *Rattus norvegicus* |
| Q58DL1 | 10 | 8 | 21 | 973 | SAGLMKR + Oxidation (M) | Arginase-2, mitochondrial | *Bos taurus* |
| P53620 | 22 | 10 | 21 | 1352 | SVPLATAPLAEQR | Coatomer subunit gamma-1 | *Bos taurus* |
| P59328 | 21 | 7 | 21 | 867 | LLAVPVEK | WD repeat and HMG-box DNA-binding protein 1 | *Mus musculus* |
| Q9EQJ9 | 13 | 8 | 21 | 1116 | DSTEQLPDGR | Membrane-associated guanylate kinase, WW and PDZ domain-containing protein 3 | *Mus musculus* |
| P26954 | 10 | 8 | 21 | 911 | EKIPNPSK | Interleukin-3 receptor class 2 subunit beta | *Mus musculus* |
| **fraction 28** | | | | | | | |
| B3EWE1 | 11 | 17 | 64  75 | 998  1793 | R.TFASFPTTK.T  K.TYFPHFDLSPGSAQVK.G | Hemoglobin subunit alpha | *Blarina brevicauda* |
| P21571 | 6 | 14 | 53  59 | 827  1061 | K.ELDPVQK.L  K.FEVLDKPQS.- | ATP synthase-coupling factor 6, mitochondrial | *Rattus norvegicus* |
| P14841 | 2 | 7 | 43 | 1207 | K.GTHTLTKSSCK.N | Cystatin-C | *Rattus norvegicus* |
| Q8VHN7 | 18 | 17 | 30 | 845 | SLSLSLAR | G-protein coupled receptor 98 | *Mus musculus* |
| Q3MHE8 | 9 | 8 | 27 | 982 | MRDHLIAK | Signal recognition particle receptor subunit alpha | *Bos taurus* |
| Q9CSP9 | 13 | 9 | 27 | 1115 | QEVEKLLEK | Tetratricopeptide repeat protein 14 | *Mus musculus* |
| O88351 | 23 | 10 | 26 | 1421 | MKNAMASTAQQLK | Inhibitor of nuclear factor kappa-B kinase subunit beta | *Mus musculus* |
| **P01211** | **6** | **8** | **26** | **717** | **YGGFMK + Oxidation (M)** | **Proenkephalin-A** | ***Bos taurus*** |
| Q9R002 | 10 | 8 | 26 | 1172 | KQHNINYEK | Interferon-activable protein 202 | *Mus musculus* |
| D3ZAF6 | 14 | 9 | 25 | 1115 | MASIVPLKEK | ATP synthase subunit f, mitochondrial | *Rattus norvegicus* |
| **Q61754** | **11** | **7** | **25** | **1204** | **DKSNDLMLLR** | **Kallikrein 1-related peptidase b24** | ***Mus musculus*** |
| Q5BIP7 | 14 | 8 | 24 | 1326 | VFGRYVFSPVR | Lipoyl synthase, mitochondrial | *Bos taurus* |
| **Q91V70** | **9** | **13** | **23** | **760** | **FQIPEK** | **Beta-defensin 7** | ***Mus musculus*** |
| Q2KJ64 | 13 | 7 | 22 | 1112 | GGVEEGPTVLR | Arginase-1 | *Bos taurus* |
| Q9R0T3 | 16 | 10 | 22 | 1058 | MVAPGSVRSR | DnaJ homolog subfamily C member 3 | *Rattus norvegicus* |
| **P12067** | **10** | **19** | **22** | **787** | **AWVAWR** | **Lysozyme C-1** | ***Sus scrofa*** |
| P00687 | 4 | 5 | 22 | 780 | DYVRTK | Alpha-amylase 1 | *Mus musculus* |
| Q3T0L2 | 6 | 8 | 22 | 834 | LAPSEYR | Endoplasmic reticulum resident protein 44 | *Bos taurus* |
| P59328 | 7 | 7 | 21 | 867 | LLAVPVEK | WD repeat and HMG-box DNA-binding protein 1 | *Mus musculus* |
| Q6AYU1 | 13 | 12 | 21 | 949 | YVDANLQK | Mortality factor 4-like protein 1 | *Rattus norvegicus* |
| **fraction 29** | | | | | | | |
| Q8SPJ1 | 30 | 15 | 30  40  68  65  33  31  33  69  56  29  57  44  55 | 997  770  1341  1352  743  813  875  846  1001  1368  811  1235  1236 | R.LAEPSQLLK.S  R.ALPELTK.L  K.LLNDEDPVVVTK.A  R.TMQNTSDLDTAR.C  R.LADGLQK.M  K.MVPLLNK.N  K.LLWTTSR.V  R.NLSDVATK.Q  K.QEGLESVLK.I  R.HPEAEMAQNSVR.L  R.LVQLLVK.A  R.ISEDKNPDYR.K  R.VSVELTNSLFK.H | Junction plakoglobin | *Bos taurus* |
| P21571 | 6 | 14 | 57  59 | 827  1061 | K.ELDPVQK.L  K.FEVLDKPQS.- | ATP synthase-coupling factor 6 | *Rattus norvegicus* |
| Q2KJ64 | 7 | 8 | 45  31  42 | 1112  1121  830 | R.GGVEEGPTVLR.K  K.LKELECDVK.D  K.TPEEVTR.T | Arginase-1 | *Bos taurus* |
| **Q61754** | **1** | **3** | **33** | **1204** | **K.DKSNDLMLLR.L** | **Kallikrein 1-related peptidase b24** | ***Mus musculus*** |
| P14841 | 14 | 8 | 27 | 1207 | GTHTLTKSSCK | Cystatin-C | *Rattus norvegicus* |
| Q2TA68 | 5 | 13 | 26 | 794 | WIYWK | Dynamin-like 120 kDa protein, mitochondrial | *Rattus norvegicus* |
| P00687 | 8 | 11 | 25 | 780 | DYVRTK | α-amylase 1 | *Mus musculus* |
| Q0VCX4 | 11 | 13 | 25 | 770 | AIPELTK | Catenin beta-1 | *Bos taurus* |
| **Q91V70** | **8** | **11** | **24** | **760** | **FQIPEK** | **β-defensin 7** | ***Mus musculus*** |
| Q9R002 | 13 | 10 | 24 | 1172 | KQHNINYEK | Interferon-activable protein 202 | *Mus musculus* |
| O08550 | 21 | 10 | 23 | 1342 | TSSPLRTSPQLR | Histone-lysine N-methyltransferase 2B | *Mus musculus* |
| Q9R0T3 | 12 | 8 | 22 | 1058 | MVAPGSVRSR | DnaJ homolog subfamily C member 3 | *Rattus norvegicus* |
| Q62924 | 12 | 13 | 21 | 822 | ELLFSSK | A-kinase anchor protein 11 | *Rattus norvegicus* |
| Q75NR7 | 7 | 8 | 21 | 849 | VPEPRPR | ATP-dependent DNA helicase Q4 | *Mus musculus* |
| P59328 | 7 | 7 | 21 | 867 | LLAVPVEK | WD repeat and HMG-box DNA-binding protein 1 | *Mus musculus* |
